# Functional principal component analysis and sparse-group LASSO to identify associations between biomarker trajectories and mortality among hospitalized SARS-CoV-2 infected individuals

**DOI:** 10.1186/s12874-023-02076-3

**Published:** 2023-10-28

**Authors:** Tingyi Cao, Harrison T. Reeder, Andrea S. Foulkes

**Affiliations:** 1 Department of Biostatistics, Harvard T.H. Chan School of Public Health, Boston, MA USA; 2 Biostatistics, Massachusetts General Hospital, Boston, MA USA; 3 Department of Medicine, Harvard Medical School, Boston, MA USA

**Keywords:** SARS-CoV-2, Biomarkers, Sparse group LASSO, Functional data analysis, Functional principal component analysis

## Abstract

**Background:**

A substantial body of clinical research involving individuals infected with severe acute respiratory syndrome coronavirus 2 (SARS-CoV-2) has evaluated the association between in-hospital biomarkers and severe SARS-CoV-2 outcomes, including intubation and death. However, most existing studies considered each of multiple biomarkers independently and focused analysis on baseline or peak values.

**Methods:**

We propose a two-stage analytic strategy combining functional principal component analysis (FPCA) and sparse-group LASSO (SGL) to characterize associations between biomarkers and 30-day mortality rates. Unlike prior reports, our proposed approach leverages: 1) time-varying biomarker trajectories, 2) multiple biomarkers simultaneously, and 3) the pathophysiological grouping of these biomarkers. We apply this method to a retrospective cohort of 12, 941 patients hospitalized at Massachusetts General Hospital or Brigham and Women’s Hospital and conduct simulation studies to assess performance.

**Results:**

Renal, inflammatory, and cardio-thrombotic biomarkers were associated with 30-day mortality rates among hospitalized SARS-CoV-2 patients. Sex-stratified analysis revealed that hematogolical biomarkers were associated with higher mortality in men while this association was not identified in women. In simulation studies, our proposed method maintained high true positive rates and outperformed alternative approaches using baseline or peak values only with respect to false positive rates.

**Conclusions:**

The proposed two-stage approach is a robust strategy for identifying biomarkers that associate with disease severity among SARS-CoV-2-infected individuals. By leveraging information on multiple, grouped biomarkers’ longitudinal trajectories, our method offers an important first step in unraveling disease etiology and defining meaningful risk strata.

**Supplementary Information:**

The online version contains supplementary material available at 10.1186/s12874-023-02076-3.

## Background

Since the outbreak of severe acute respiratory syndrome coronavirus 2 (SARS-CoV-2) in December 2019, more than 670 million confirmed cases and 6.8 million associated deaths have been reported worldwide, with a large proportion of these deaths preceded by hospitalization [1]. The vast amount of data collected and stored in electronic health records among hospitalized patients provides an opportunity to identify early predictors of severe disease. Ultimately understanding the relationship between patient level in-hospital data, including early biomarker trajectories, and severe outcomes may inform disease etiology, risk stratification, and resource allocation.

Among SARS-CoV-2 infected individuals, multiple biomarkers are typically measured repeatedly over the duration of hospitalization. An extensive literature has identified correlations between biomarker levels and severe outcomes, including intubation, admission to intensive care units, and death among hospitalized SARS-CoV-2 infected individuals [2–14]. However, most existing studies considered each biomarker independently [2–9] with a few exceptions that applied penalized regression and other machine learning techniques [10, 11]. None of these manuscripts, to our knowledge, accounted for the pathophysiological relationships among biomarkers. Moreover, repeatedly measured biomarkers were typically reduced to baseline or peak values [2–8, 10, 11], with again a small number of exceptions, including one report using linear mixed-effects models to account for the entire biomarker trajectory [9]. To our knowledge, analyses that simultaneously consider multiple biomarkers as well as their longitudinal trajectories in evaluating associations with severe SARS-CoV-2 outcomes have not been reported.

Methods for joint modeling of multiple longitudinal biomarkers and time-to-event outcomes have also been described [15–18]. However, as these methods generally require computationally intensive procedures, such as multi-dimensional numerical integration or complex Bayesian sampling schemes, approaches incorporating variable selection among multiple biomarkers into the joint modeling framework remain limited [18, 19]. A scalable alternative involves application of multivariate functional principal component analysis (FPCA) [20] to reduce each biomarker trajectory to a set of scores and then using these scores as covariates in a survival model [21–23]. Application of FPCA and survival modeling has been limited to prediction of the time-to-event outcome. To allow for variable selection in this context, we propose a two-stage analytic strategy that combines FPCA and sparse-group LASSO (SGL) [24], abbreviated as FPCA-SGL, to characterize associations between multiple biomarker trajectories and mortality, while also leveraging the pathophysiological grouping of these biomarkers.

## Methods

### Study population

Data derived from a retrospective cohort of 12,941 patients infected with SARS-CoV-2 based on hospital record ICD-10 codes (U07.1, B34,2, and B97.29) and positive PCR tests between March 1, 2020 and November 30, 2021 were used for analysis (Table 1). All patients were hospitalized at Massachusetts General Hospital or Brigham and Women’s Hospital (MGB) within 5 days prior to and 30 days after a positive SARS-CoV-2 test. Patients hospitalized for less than 24 hours or with an unknown duration were excluded.

**Table 1:** Demographic characteristics, survival outcomes and time in hospitalization stratified by sex

	Female (n=6300)	Male (n=6641)	Total (n=12941)
Age			
Age$\le$50	2138 (33.9%)	2007 (30.2%)	4145 (32.0%)
50<Age$\le$65	1530 (24.3%)	2061 (31.0%)	3591 (27.7%)
Age>65	2632 (41.8%)	2573 (38.7%)	5205 (40.2%)
Race			
White, non-Hispanic	3610 (57.3%)	3961 (59.6%)	7571 (58.5%)
Black, non-Hispanic	820 (13.0%)	698 (10.5%)	1518 (11.7%)
Asian, non-Hispanic	213 (3.4%)	251 (3.8%)	464 (3.6%)
Hispanic	1317 (20.9%)	1341 (20.2%)	2658 (20.5%)
Other/Unknown	340 (5.4%)	390 (5.9%)	730 (5.6%)
BMI			
Normal (BMI<25)	1368 (21.7%)	1341 (20.2%)	2709 (20.9%)
Overweight (25$\le$BMI<30)	1528 (24.3%)	2050 (30.9%)	3578 (27.6%)
Obese (BMI$\ge$30)	2502 (39.7%)	2132 (32.1%)	4634 (35.8%)
Missing	902 (14.3%)	1118 (16.8%)	2020 (15.6%)
Deaths			
Number of deaths	488 (7.7%)	710 (10.7%)	1198 (9.3%)
Time (days) to death	10.5 (5, 16)	11 (6, 18)	11 (6, 17)
Time (days) in hospital			
Overall	5 (3, 9)	5 (3, 10)	5 (3, 10)
For dead within 30 days	8 (4, 13)	8 (5, 15)	8 (4, 14)
For alive and discharged within 30 days	5 (3, 8)	5 (3, 8)	5 (3, 8)
For alive and still in hospital after 30 days	38.5 (33, 46.25)	41 (34, 54)	39.5 (34, 52.5)

Summary measures are presented as count (percentage) for categorical variables and median (interquartile range) for continuous variables

### Data pre-processing

The primary outcome is 30-day mortality since hospital admittance. Death records from both the MGB Enterprise Data Warehouse (EDW) and the Massachusetts Registry of Vital Records and Statistics were obtained, and in the case of an inconsistency between the two sources, death dates from the Registry were adopted. Exposures are repeated laboratory measurements of $m=20$ routine biomarkers collected during hospitalization up to 30 days. Biomarker data were extracted from MGB EDW and if there were multiple measurements of a biomarker for one patient within a 24-hour period, the mean value of the measurements was used. Censored laboratory measurements were treated as known at the cut-off value. Demographic information including age, sex, race/ethnicity and body mass index (BMI) was obtained from MGB EDW. Biomarkers were divided into six categories based on their pathophysiological functions, as shown in Supplementary Table 1.

### Statistical analysis

A two-stage analytic approach was considered. First, FPCA was performed separately on each of the $k=1,2,\cdots ,K$ biomarkers. Each biomarker’s repeated measurements were treated as functional data, i.e., independent realizations of a smooth random function $X_k(t)$ [20]. Through spectral decomposition of the covariance operator, FPCA reduces the functional data into eigenfunctions $\phi _{km}(t)$ for $m=1,\cdots ,M$, referred to as functional principal components (FPCs). Each individual *i* has a set of coefficients for these eigenfunctions called FPC scores, denoted as $A_{kim}$. Thus the trajectory of one biomarker for patient *i*, $X_{ki}(t)$, can be expanded as

$$\begin{aligned} X_{ki}(t) = \mu _k(t) + \sum _{m=1}^\infty A_{kim}\phi _{km}(t) \approx \mu _k(t) + \sum _{m=1}^M A_{kim}\phi _{km}(t), \end{aligned}$$

where $\mu _k(t)=\mathbb {E}[X_k(t)]$ is the mean function. Then each patient’s FPC scores, $A_{kim}, m=1,\cdots ,M$, characterize the variation of individual level biomarker trajectories from the sample mean function. We adopted the PACE method which computes the FPC scores as conditional expectations because it is suitable for sparse and irregularly spaced longitudinal data like our biomarker data [25]. To implement FPCA using the PACE approach, we used the fdapace package in R [26]. Based on the cumulative percentage of variance explained, we determined the number of FPCs (*M*) to adopt, resulting in $K\times M$ exposure variables. Missing FPC scores were imputed using MICE based on the FPC scores of all other biomarkers [27].

Second, using the SGL package in R, we performed Cox SGL regression with the $K\times M$ FPC scores as the exposure variables, while adjusting for *J* demographic characteristics $Y_{ij}$ including race/ethnicity, age (indicator for $>50$ years), and BMI (orthogonal polynomials of degree 2):

$$\begin{aligned} h_i(t) = h_0(t) \text {exp} \left[ \left( \sum _{k=1}^K \sum _{m=1}^M \delta _{km}A_{kim} \right) + \sum _{j=1}^J \zeta _jY_{ij}\right] , \end{aligned}$$

where there are $K\times M+J$ regression parameters to be estimated, collectively denoted as $\varvec{\beta }=\{\delta _{km},\zeta _j\}$ for $k=1,\cdots ,K$, $m=1,\cdots ,M$, and $j=1,\cdots ,J$.

SGL estimates the $\varvec{\beta }$’s using a weighted combination (controlled by a hyperparameter $\eta$) of group LASSO $l_1$-penalty term and the standard parameter-wise LASSO $l_1$-penalty term to induce both groupwise and within-group sparsity [24]:

$$\begin{aligned} (1-\eta )\lambda \sum _{l=1}^L \sqrt{p_l} \left\| \beta ^{(l)}\right\| _2 + \eta \lambda \Vert \beta \Vert _{1} \end{aligned}$$

where the grouping $l=1,\cdots ,L$ represents biomarkers’ six pathophysiological categories and one group for all demographic characteristics, i.e. for $l=1,2,\cdots ,6$, $\beta ^{(l)} = \{ \delta _{km}\}$ for *k* over all biomarkers in that pathophysiological group and $m=1,\cdots ,M$ for each biomarker’s *M* FPC scores; and $\beta ^{(7)} = \{ \zeta _j\}$ for $j=1,\cdots ,J$.

This two-stage strategy, FPCA decomposition of the biomarker trajectoris followed by SGL, allowed us to identify biomarkers associated with 30-day mortality while accounting for within-group correlations between biomarkers as well as the time varying biomarker trajectories. Both stages of analysis were stratified by sex.

We selected the overall regularization parameter $\lambda$ through a 10-fold cross validation (CV) from a pre-specified sequence of 100 $\lambda$ values. The sequence of 100 candidate $\lambda$ values were chosen such that the maximum, $\lambda _\text {max}$, was the smallest possible $\lambda$ that shrunk all coefficients to zero, the minimum, $\lambda _\text {min}$, was set equal to $\lambda _\text {max}/100$, and all other $\lambda$ values were spaced equally between $\lambda _{\text {min}}$ and $\lambda _{\text {max}}$. Eventually, we selected $\lambda _\text {1se}$ which was the largest value of $\lambda$ such that the CV error, defined as the CV negative log likelihood, was within 1 standard error of the minimum.

We set the weight for the group LASSO and LASSO penalty terms to $1-\eta = 0.7$ and $\eta = 0.3$, respectively to get a group LASSO structure with limited within-group sparsity. This reflects that biomarkers are expected to exhibit group structure due to their underlying pathophysiological relationships, while still allowing individual biomarkers or FPC scores to be excluded from the model to enhance sparsity. Alternatively, this weight parameter $\eta$ could be tuned via an additional layer of CV. As a sensitivity analysis, we fit the SGL model with different weights, $\eta = 0.05, 0.50, 0.70, 0.95$ respectively, to examine whether the results were sensitive to the choice of this hyperparameter.

For comparison, we considered application of SGL using only the baseline or peak measurements of each of the *K* biomarkers in place of the FPC scores. Here we log transformed the baseline and peak measurements to ensure they were approximately normally distributed, and again imputed missing values with available biomarker measures using the MICE package in R [27]. Analyses were again stratified by sex and adjusted for race/ethnicity, age, and BMI while accounting for the pathophysiological groupings of biomarkers.

### Simulation studies

To characterize the performance of our proposed two-stage approach, we conducted simulation studies including 200 repetitions with sample sizes of $n=2000$ for each condition [21, 28] (Supplementary Methods). We first simulated trajectories of four biomarkers belonging to two groups, denoted as $Z_{ki}(t)$ where $k=1,2,3,4$ and $i=1,2,\cdots ,n$. Biomarkers $k=1,2$ were in the first group with relatively low within-group correlation, and biomarkers $k=3,4$ were in the second group with relatively high within-group correlation. $Z_{ki}(t)$ were simulated under three models: Model 1 was a linear mixed-effects model (LME) with a linear time trend; Model 2 was a LME with a quadratic term for time; and Model 3 was a LME with a 3-knot spline function for time.

Death times were simulated based on $Z_{ki}(t)$ using inverse transform sampling on the survival function derived from the following hazard function: $h_i(t) = h_0(t) \text {exp} \left[ \alpha _1\times Z_{1i}(t) + \alpha _2\times Z_{2i}(t) + \alpha _3\times Z_{3i}(t) + \alpha _4\times Z_{4i}(t) \right]$. The association parameters $\alpha _1,\alpha _2,\alpha _3,\alpha _4$, were specified based on four scenarios: Scenario 1, only the low correlation biomarker group was associated with mortality; Scenario 2, only the high correlation biomarker group was associated with mortality; Scenario 3, both biomarker groups were associated with mortality; Scenario 4, neither of the two biomarker groups was associated with mortality (Table 2). Measurement error was added to the true trajectories to simulate observed trajectories. Lastly, we simulated censoring by truncating observed trajectories at the patient’s time of death or discharge (Supplementary Methods).

**Table 2:** Set up of simulation studies

	$\varvec{\alpha }_{\varvec{1}}$	$\varvec{\alpha }_{\varvec{2}}$	$\varvec{\alpha }_{\varvec{3}}$	$\varvec{\alpha }_{\varvec{4}}$
Model 1 (LME with a linear time trend)				
Scenario 1 (low correlation)	1	1	0	0
Scenario 2 (high correlation)	0	0	1	1
Scenario 3 (both groups)	1	1	1	1
Scenario 4 (null case)	0	0	0	0
Scenario 5^a^ (complete null case)	0	0	0	0
Model 2 (LME with a quadratic term for time)				
Scenario 1 (low correlation)	1	1	0	0
Scenario 2 (high correlation)	0	0	1	1
Scenario 3 (both groups)	0.5	0.5	0.5	0.5
Scenario 4 (null case)	0	0	0	0
Scenario 5^a^ (complete null case)	0	0	0	0
Model 3 (LME with a 3-knot spline function for time)				
Scenario 1 (low correlation)	1	1	0	0
Scenario 2 (high correlation)	0	0	1	1
Scenario 3 (both groups)	1	1	1	1
Scenario 4 (null case)	0	0	0	0
Scenario 5^a^ (complete null case)	0	0	0	0

^a^Scenario 5 is an additional scenario based on Scenario 4 where we did not censor biomarker trajectories by death times. Details about this scenario were explained in the Results section

For the primary analysis we applied FPCA-SGL using the top three FPC scores ($M=3$) for each biomarker as the exposure variables, i.e. $K\times M=4\times 3=12$ variables. The weights for the group LASSO and LASSO penalty terms were set to $1-\eta =0.95$ and $\eta =0.05$, respectively because our simulated scenarios were a group LASSO case. To assess performance of our proposed approach, we reported the true positive rate (TPR), defined as the proportion of simulations where truly non-zero coefficients were selected, and the false positive rate (FPR), defined as the proportion of simulations where truly zero coefficients were selected. We also compared FPCA-SGL to two simpler comparator approaches using baseline or peak measurements alone.

## Results

### Application using MGB cohort

The MGB cohort was composed of 12,941 patients, $32\%$ were $\le 50$ years of age, $20.5\%$ Hispanic, $11.7\%$ Black/non-Hispanic, and $35.8\%$ obese (BMI$\ge 30$) (Table 1). 1,198 patients ($9.3\%$) died within 30-days of hospitalization, with a higher proportion in males than in females ($10.7\%$ vs. $7.7\%$) (Table 1). Supplementary Table 1 summarizes laboratory measurements for 20 biomarkers. More than $60\%$ of patients had at least one measurement on each of the 20 biomarkers, with a median number of measurements for each biomarker ranging from 1 to 6, except that d-dimer had fewer measurements.

We performed FPCA on each of the 20 biomarkers, stratified by sex. Supplementary Fig. 1 displayed the mean function and corresponding FPCs of each biomarker. Across the 20 biomarkers, the first three FPCs cumulatively explained a median of $97.39\%$ [IQR = ($95.32\%$, $99.22\%$)] and $97.49\%$ [IQR = ($96.06\%$, $98.48\%$)] of the total variance among females and males, respectively. Therefore, we picked $M=3$ FPCs for each of the *M* biomarkers. FPC scores were approximately normally distributed (Supplementary Fig. 2).

To better illustrate how each patient’s $M=3$ FPC scores could represent the variation of their individual biomarker trajectories from the mean function, Supplementary Fig. 3 plotted the trajectories of blood urea nitrogen (bun) of three male patients with different FPC scores $A_{k=1,i=\{1,2,3\},m=\{1,2,3\}}$. It shows how each individual’s trajectory decomposes into a linear combination of the mean function and three eigenfunctions, resulting in different individual-specific FPC scores.

**Fig. 1:**
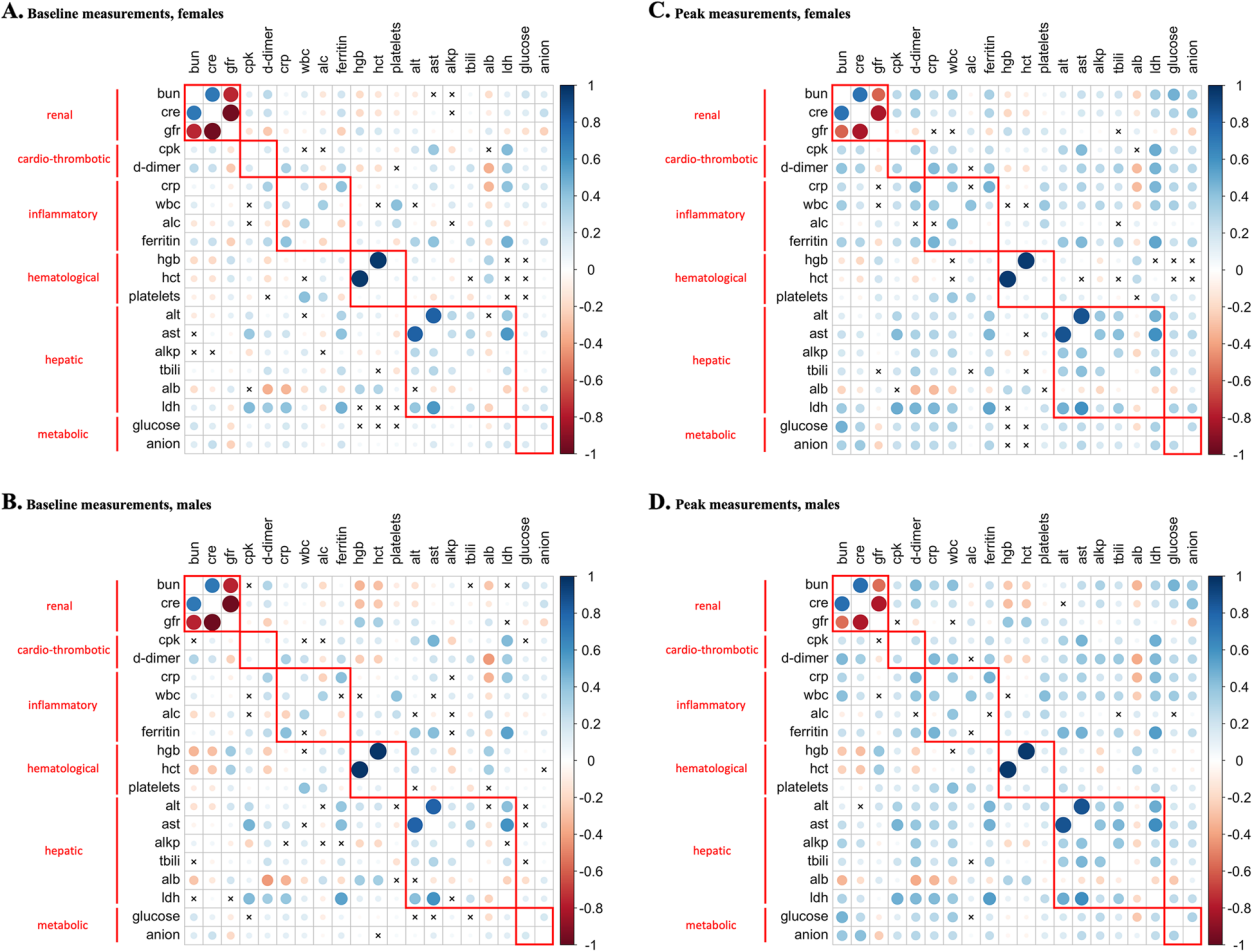
Pearson correlations between biomarkers’ baseline and peak measurements, stratified by sex and masked by *p*-value under an $\alpha -$level of 0.05

The pairwise baseline biomarker correlations were similar among females and males (Fig. 1A, B). The renal, hematological, and the hepatic groups exhibited high within-group correlation while the cardio-thrombotic, inflammatory and metabolic groups presented low within-group correlation. The across-group correlations were generally low (Fig. 1A, B). The pairwise peak biomarker correlations showed similar patterns (Fig. 1C, D). Biomarker peak and baseline values were approximately normally distributed after log transformation, imputation and standardization, with the exception of estimated glomerular filtration rate and total bilirubin (Supplementary Fig. 4).

**Fig. 2:**
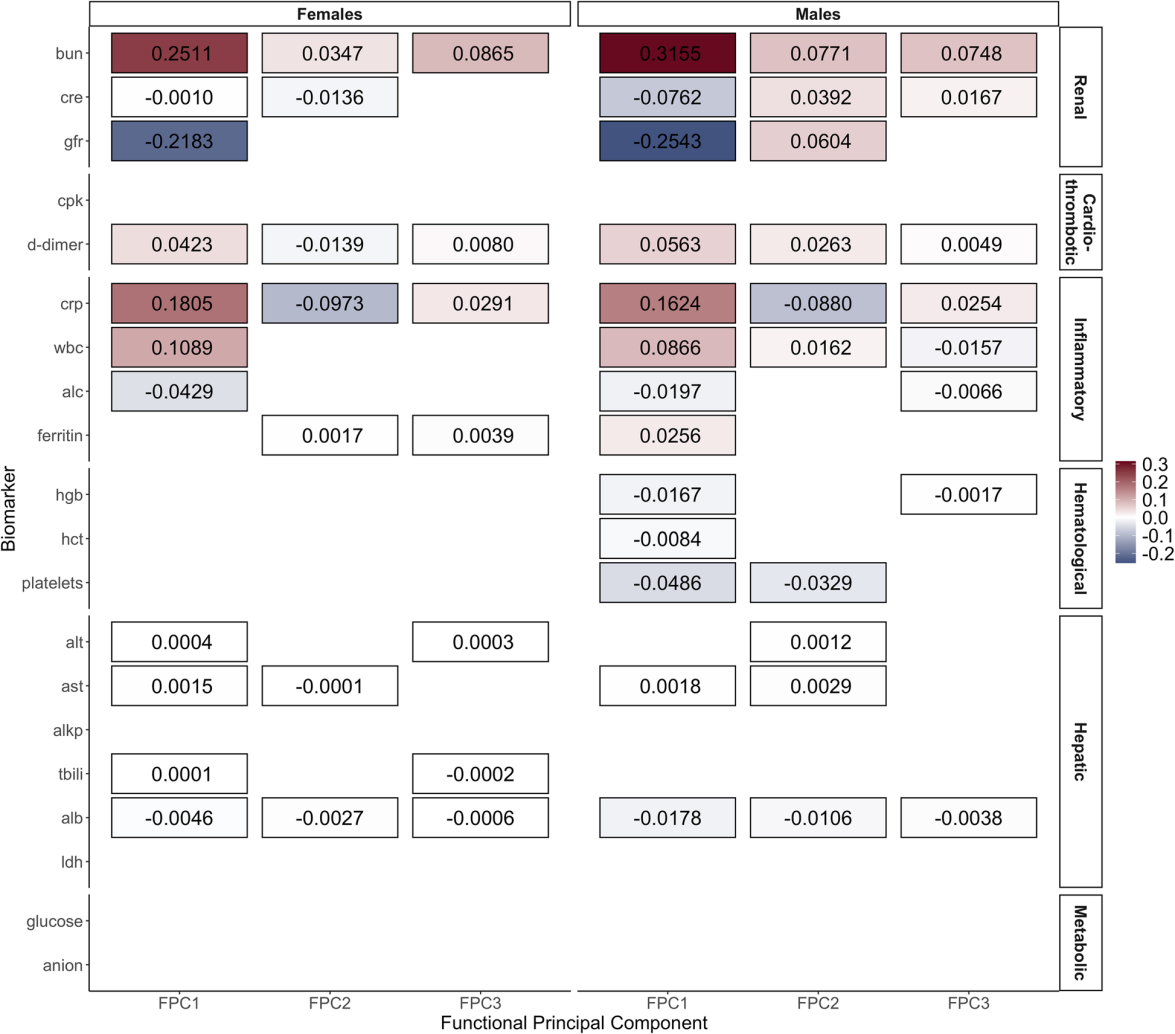
Estimated regression coefficients $\hat{\varvec{\beta }}$ from SGL models fitted with the scores of the first 3 FPCs of each biomarker as exposure variables, tiles with no border or annotated numbers indicate $\hat{\varvec{\beta }}$ being regularized to zero (The full names of the abbreviated biomarkers are listed at the end of the manuscript)

Using the FPCA-SGL approach with $K\times M=20\times 3=60$ FPC scores as exposure variables, we found biomarkers in the renal and inflammatory groups to be strongly associated with mortality in both males and females. In the cardio-thrombotic group, only d-dimer appeared to be associated with mortality. Biomarkers in the hepatic groups showed slight associations while the metabolic group was not associated with mortality. The hematological group was associated with mortality among males but not females (Fig. 2).

**Fig. 3:**
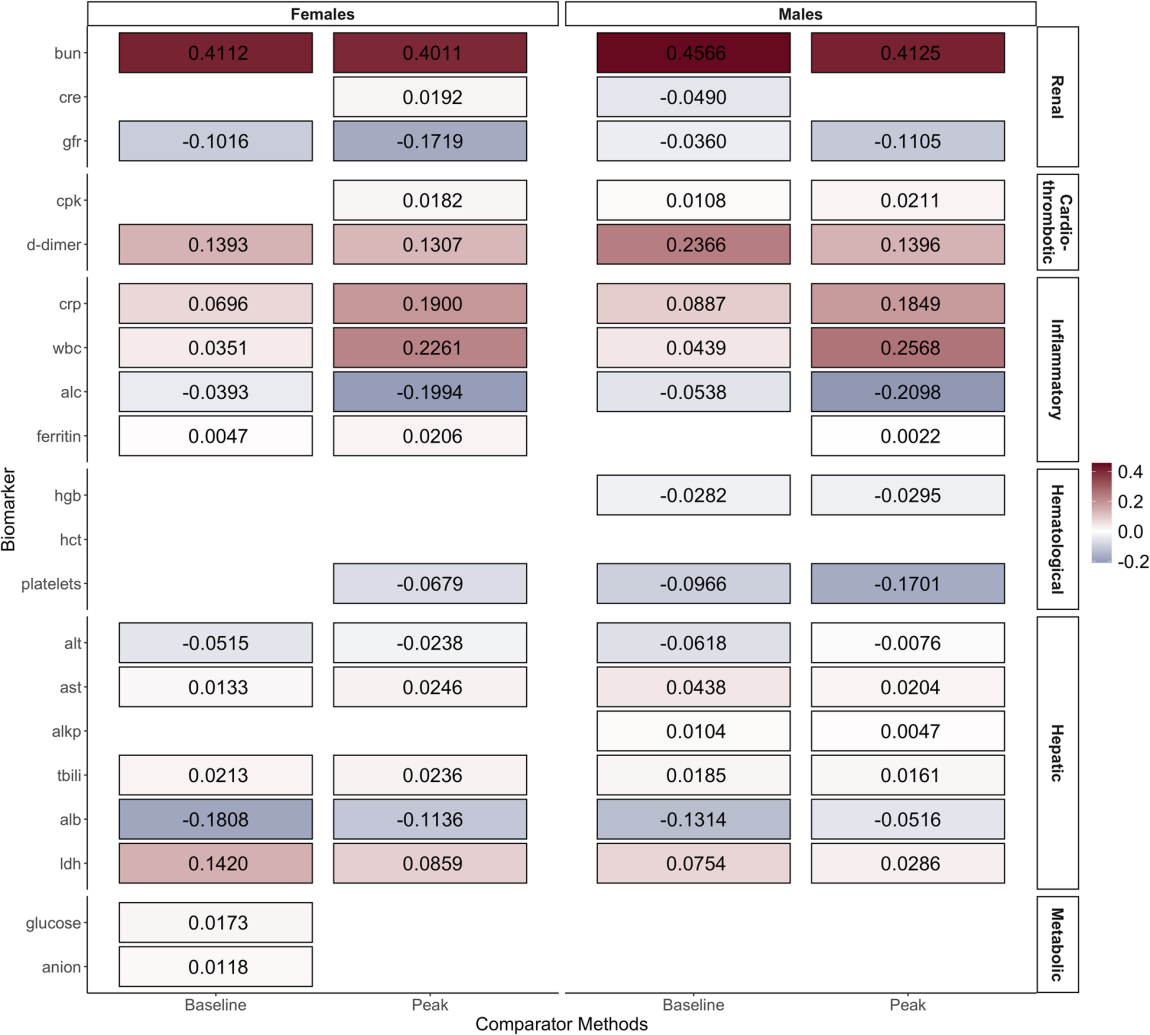
Estimated regression coefficients $\hat{\varvec{\beta }}$ from SGL models fitted with the baseline or peak measurement of each biomarker as exposure variables, tiles with no border or annotated numbers indicate $\hat{\varvec{\beta }}$ being regularized to zero (The full names of the abbreviated biomarkers are listed at the end of the manuscript)

For comparison, we used each of the 20 baseline measurements and the 20 peak measurements as exposure variables, and fitted SGL Cox regressions stratified by sex. Using baseline measurement, all biomarker groups except for the hematological group among females and the metabolic group among males were associated with mortality, with some degree of within-group sparsity observed (Fig. 3). Using peak measurements, most biomarkers across all groups except for the metabolic group were associated with mortality, with almost no within-group sparsity (Fig. 3). Results from our simulation studies, as presented below, demonstrated that using baseline or peak measurements can result in high false positive rates.

As a sensitivity analysis, we applied the FPCA-SGL approach with the same 60 FPC scores while changing the weight hyperparameter $\eta$, with larger $\eta$ implying more LASSO than group LASSO structure. Supplementary Fig. 5 displayed four similar heatmaps as Fig. 2 for four different weight values: $\eta =0.05, 0.50, 0.70, 0.95$. The associations were similar with the main analysis: the renal and inflammatory groups were still strongly associated with mortality in both sexes; in the cardio-thrombotic group, d-dimer showed associations and creatine phosphokinase only showed trivial associations when a group LASSO structure was enforced under $\eta <0.3$; the hepatic group was slightly associated with mortality and under small $\eta$ the entire group was not selected, while larger $\eta$ revealed that the aspartate aminotransferase and albumin in the group were the biomarkers driving these associations; the metabolic group was not associated with mortality; the hematological group showed associations still only among males but not females. Therefore, this analysis demonstrated that the results were not sensitive to the choice of the weight hyperparameter.

### Simulation studies

Both our proposed two-stage FPCA-SGL method and the simpler comparator methods using baseline or peak measurements, offered high TPR, i.e., high sensitivity, under Scenario 1-3 for Model 1-3: the approach using baseline measurements always gave TPR as high as $100\%$ and never smaller than $98.5\%$; the approach using peak measurements always gave TPR as high as $100\%$ and never smaller than $98\%$; our proposed approach using FPC scores gave comparable TPR over $98.5\%$ for all first FPCs and relatively high TPR greater than $87\%$ for the second and third FPCs (Table 3).

**Table 3:** Results of simulation studies

	Scenario 1		Scenario 2		Scenario 3	Scenario 4
	TPR	FPR	TPR	FPR	TPR	FPR
A. Model 1 (LME with a linear time trend)						
A.1 Using baseline measures						
Group 1						
Biomarker 1	100.0%	N/A	N/A	10.0%	100.0%	14.0%
Biomarker 2	100.0%	N/A	N/A	10.0%	100.0%	14.5%
Group 2						
Biomarker 3	N/A	6.5%	100.0%	N/A	100.0%	15.0%
Biomarker 4	N/A	6.5%	100.0%	N/A	100.0%	16.0%
A.2 Using peak measures						
Group 1						
Biomarker 1	100.0%	N/A	N/A	5.0%	100.0%	18.0%
Biomarker 2	100.0%	N/A	N/A	5.0%	100.0%	17.5%
Group 2						
Biomarker 3	N/A	12.0%	100.0%	N/A	100.0%	17.0%
Biomarker 4	N/A	12.0%	100.0%	N/A	100.0%	17.0%
A.3 Using FPC scores						
Group 1						
Biomarker 1 FPC1	100.0%	N/A	N/A	0.0%	100.0%	9.0%
Biomarker 1 FPC2	94.0%	N/A	N/A	0.0%	92.0%	9.0%
Biomarker 1 FPC3	90.5%	N/A	N/A	0.0%	92.5%	9.0%
Biomarker 2 FPC1	100.0%	N/A	N/A	0.0%	100.0%	8.5%
Biomarker 2 FPC2	93.5%	N/A	N/A	0.0%	93.5%	9.0%
Biomarker 2 FPC3	90.5%	N/A	N/A	0.0%	88.0%	9.0%
Group 2						
Biomarker 3 FPC1	N/A	3.5%	100.0%	N/A	100.0%	5.5%
Biomarker 3 FPC2	N/A	3.0%	92.5%	N/A	91.5%	5.5%
Biomarker 3 FPC3	N/A	3.5%	91.0%	N/A	93.0%	5.5%
Biomarker 4 FPC1	N/A	3.5%	100.0%	N/A	100.0%	5.5%
Biomarker 4 FPC2	N/A	3.5%	89.5%	N/A	93.0%	5.5%
Biomarker 4 FPC3	N/A	3.5%	91.5%	N/A	87.0%	5.5%
B. Model 2 (LME with a quadratic term for time)						
B.1 Using baseline measures						
Group 1						
Biomarker 1	100.0%	N/A	N/A	21.0%	99.0%	15.5%
Biomarker 2	100.0%	N/A	N/A	21.0%	99.0%	15.5%
Group 2						
Biomarker 3	N/A	18.0%	100.0%	N/A	100.0%	17.0%
Biomarker 4	N/A	18.0%	100.0%	N/A	100.0%	17.5%
B.2 Using peak measures						
Group 1						
Biomarker 1	100.0%	N/A	N/A	22.0%	98.0%	14.0%
Biomarker 2	100.0%	N/A	N/A	22.0%	98.0%	13.5%
Group 2						
Biomarker 3	N/A	17.0%	100.0%	N/A	100.0%	17.5%
Biomarker 4	N/A	17.0%	100.0%	N/A	100.0%	18.0%
B.3 Using FPC scores						
Group 1						
Biomarker 1 FPC1	100.0%	N/A	N/A	0.0%	98.5%	12.0%
Biomarker 1 FPC2	98.0%	N/A	N/A	0.0%	95.0%	12.0%
Biomarker 1 FPC3	90.0%	N/A	N/A	0.0%	87.5%	11.5%
Biomarker 2 FPC1	100.0%	N/A	N/A	0.0%	98.5%	12.0%
Biomarker 2 FPC2	96.0%	N/A	N/A	0.0%	89.5%	12.0%
Biomarker 2 FPC3	87.5%	N/A	N/A	0.0%	87.0%	11.5%
Group 2						
Biomarker 3 FPC1	N/A	0.5%	100.0%	N/A	100.0%	10.5%
Biomarker 3 FPC2	N/A	0.5%	97.0%	N/A	95.0%	10.5%
Biomarker 3 FPC3	N/A	0.5%	90.0%	N/A	90.0%	10.5%
Biomarker 4 FPC1	N/A	0.5%	100.0%	N/A	100.0%	10.5%
Biomarker 4 FPC2	N/A	0.5%	97.0%	N/A	90.5%	10.0%
Biomarker 4 FPC3	N/A	0.5%	89.0%	N/A	92.5%	10.5%
C. Model 3 (LME with a 3-knot spline function for time)						
C.1 Using baseline measures						
Group 1						
Biomarker 1	100.0%	N/A	N/A	5.5%	98.5%	16.5%
Biomarker 2	100.0%	N/A	N/A	5.5%	98.5%	17.5%
Group 2						
Biomarker 3	N/A	5.5%	100.0%	N/A	100.0%	16.0%
Biomarker 4	N/A	5.5%	100.0%	N/A	100.0%	16.0%
C.2 Using peak measures						
Group 1						
Biomarker 1	100.0%	N/A	N/A	5.0%	99.5%	41.5%
Biomarker 2	100.0%	N/A	N/A	5.0%	99.5%	40.5%
Group 2						
Biomarker 3	N/A	10.0%	100.0%	N/A	100.0%	40.5%
Biomarker 4	N/A	10.0%	100.0%	N/A	100.0%	40.5%
C.3 Using FPC scores						
Group 1						
Biomarker 1 FPC1	100.0%	N/A	N/A	0.0%	99.0%	9.0%
Biomarker 1 FPC2	95.0%	N/A	N/A	0.0%	89.5%	8.5%
Biomarker 1 FPC3	91.0%	N/A	N/A	0.0%	89.0%	8.5%
Biomarker 2 FPC1	100.0%	N/A	N/A	0.0%	99.0%	8.5%
Biomarker 2 FPC2	94.0%	N/A	N/A	0.0%	93.5%	8.5%
Biomarker 2 FPC3	95.5%	N/A	N/A	0.0%	90.5%	8.5%
Group 2						
Biomarker 3 FPC1	N/A	3.5%	100.0%	N/A	100.0%	8.0%
Biomarker 3 FPC2	N/A	4.0%	94.5%	N/A	93.5%	8.0%
Biomarker 3 FPC3	N/A	4.0%	88.5%	N/A	88.5%	8.0%
Biomarker 4 FPC1	N/A	4.0%	100.0%	N/A	100.0%	8.0%
Biomarker 4 FPC2	N/A	4.0%	96.5%	N/A	92.5%	7.5%
Biomarker 4 FPC3	N/A	4.0%	88.0%	N/A	88.5%	7.5%

True positive rate (TPR) calculated as the proportion of simulations where truly non-zero coefficients were selected, or false positive rate (FPR) calculated as the proportion of simulations where truly zero coefficients were selected

The FPCA-SGL approach gave relatively low FPR, as low as $0\%$ in Scenario 2 in the case that the biomarker group with high within-group correlation was associated with the survival outcome, and no higher than $12\%$ in the null case of Scenario 4. Notably, in every scenario under every model, this approach consistently showed smaller FPR than the approaches using baseline or peak measurements, especially under Scenario 1-2 for Model 2 (FPR ranging from $17\%$ to $22\%$ using baseline or peak measurements but as low as $0-0.5\%$ using FPC scores). This demonstrated that our proposed two-stage FPCA-SGL approach gave much higher specificity than the simpler methods using baseline or peak measurements (Table 3).

The two comparator methods, especially the one using peak measurements, suffered from high FPR, which was particularly high in Scenario 4: the approach using peak measurements yielded an FPR of $17-18\%$ under Model 1, $13.5-18\%$ under Model 2, and $40.5-41.5\%$ under Model 3; the approach using baseline measurements yielded an FPR of $14-16\%$ under Model 1, $15.5-17.5\%$ under Model 2, and $16.0-17.5\%$ under Model 3 (Table 3). This again illustrated that these two simpler approaches using baseline or peak measurements suffered from low specificity.

To investigate further the inflated FPR in Scenario 4, we considered an additional Scenario 5, a complete null case in which neither of the two biomarker groups was associated with mortality ($\alpha _1=\alpha _2=\alpha _3=\alpha _4=0$) and observed biomarker trajectories were not censored by death times (Table 2). Simulation results showed that the FPR decreased slightly in this scenario, most notably under Model 3 (from $40.5-41.5\%$ in Scenario 4 to $16.5-20\%$ in Scenario 5 with peak measurements, from $7.5-9\%$ in Scenario 4 to $5-6.5\%$ in Scenario 5 with FPC scores), and also under Model 1 with peak measurements (from $17-18\%$ in Scenario 4 to $13.5-16\%$ in Scenario 5), as well as under Model 2 with FPC scores (from $10-12\%$ in Scenario 4 to $4-5\%$ in Scenario 5) (Supplementary Table 2). For the approach using baseline measurements, this additional Scenario 5 did not alter the baseline values thus the FPR remained similar (Supplementary Table 2).

## Discussion

Our proposed FPCA-SGL approach revealed associations between several biomarker trajectories and 30-day mortality among hospitalized SARS-CoV-2 patients. In particular, renal and inflammatory biomarkers were strongly associated with mortality risks. Several studies have examined incidence of acute kidney injury (AKI) among SARS-CoV-2 patients and discovered that AKI was related to more severe outcomes including death, respiratory failure, and disseminated intravascular coagulation [7, 10, 29]. Elevated blood urea nitrogen and creatinine, as well as lower estimated glomerular filtration rate were all markers of AKI and were reported to be correlated with worse outcomes [2, 5, 7, 10, 13, 29]. Studies have indicated excessive inflammatory response as a contributory factor to SARS-CoV-2 disease severity [30]. Lymphocytes are crucial in modulating inflammatory response and maintaining immune homeostasis during viral infection [31], and research reported elevated white blood cell count and lymphopenia (low absolute lymphocyte count) among severe SARS-CoV-2 patients [3, 4, 7]. Elevated c-reactive protein levels were also closely related to inflammation and shown to be highly associated with disease severity [32].

A limited number of studies have specifically investigated the effect of sex on the associations between biomarker levels and disease severity [12–14]. We had a relatively large cohort of 12,941 patients, thus we conducted our analyses under stratification by sex so as to better explore any potential sex modification. Interestingly, we observed associations of hematological biomarkers (hemoglobin, hematocrit, and platelets) with 30-day mortality risks only among males. As males usually experienced more severe symptoms and worse survival outcomes during SARS-CoV-2 infection [12, 14], our results may lend insight into the sex difference behind the cellular and molecular pathways underlying SARS-CoV-2 disease progression.

Methodologically, our proposed FPCA-SGL approach is an easy-to-implement and computationally efficient analytic strategy that is able to simultaneously consider multiple biomarkers as well as their longitudinal trajectories in evaluating associations with severe SARS-CoV-2 outcomes. It is a versatile alternative to existing methods concerning multiple longitudinal measurements and a survival outcome and could be applied in other areas. Using simulation studies, we demonstrated that FPCA-SGL retained high TPR and outperformed alternative approaches using baseline or peak values with respect to FPR. In particular, we observed a substantial “survival bias” (inflated FPR) in our simulations when using peak measurements, because they are endogenous covariates, meaning their values and future paths are directly affected by the survival outcome of interest [33]. For example, if a certain biomarker has a monotonically increasing trajectory during hospitalization, the peak value observed will be higher for patients surviving longer, causing spurious associations between lower peak biomarker values and higher mortality risks. This “survival bias” resulted in high FPR in our simulation study using the peak measurement approach (Scenario 4 in Table 3) and was moderately alleviated when we did not censor the simulated biomarker trajectories based on simulated death times (Scenario 5 in Supplementary Table 2). Our FPCA-SGL approach mitigated this “survival bias” (lower FPR in Scenario 4 in Table 3) because FPCA naturally imputed biomarkers’ unobserved future trajectories even after patients’ deaths. Nonetheless, we did still observe some false positives using this proposed approach with FPC scores (largest FPR as $12\%$ in Scenario 4 in Table 3).

## Conclusions

We presented a two-stage analytic approach that combined FPCA and SGL to study the associations between hospitalized SARS-CoV-2 patients’ multiple biomarker trajectories with their 30-day mortality rates. We demonstrated that this method had high TPR and outperformed simpler comparator approaches using biomarkers’ baseline or peak measurements with respect to FPR. Using data from a retrospective cohort of 12,941 patients, we showed that renal biomarkers (blood urea nitrogen, creatinine, and estimated glomerular filtration rate), inflammatory biomarkers (c-reactive protein, white blood cell count, and absolute lymphocyte count), cardio-thrombotic biomarkers (d-dimer) were associated with 30-day mortality rates among hospitalized SARS-CoV-2 patients. Our sex-stratified analysis also revealed that hematological biomarkers (hemoglobin, hematocrit, and platelets) were associated with higher mortality only among males. This study recognized the prognostic value of biomarkers as well as the underlying potential sex difference. These results provide insights into assessment of SARS-CoV-2 disease severity and effective risk stratification.

### Supplementary Information

**Additional file 1.** Supplementary Figures and Tables.

**Additional file 2.** Supplementary Methods.

## Data Availability

The data is not publicly available. The code for the simulation studies (both data simulation and the application of our proposed FPCA-SGL method) is available in the Github repository, https://github.com/Aimeessn/FPCA-SGL.

## Abbreviations

bun: Blood urea nitrogen
cre: Creatinine
gfr: Estimated glomerular filtration rate
cpk: Creatine phosphokinase
crp: C-reactive protein
wbc: White blood cell count
alc: Absolute lymphocyte count
hgb: Hemoglobin
hct: Hematocrit
alt: Alanine aminotransferase
ast: Aspartate aminotransferase
alkp: Alkaline phosphatase
tbili: Total bilirubin
alb: Albumin
ldh: Lactate dehydrogenase
anion: Anion gap

## References

[1] Dong E, Du H, Gardner L (2020). An interactive web-based dashboard to track COVID-19 in real time. Lancet Infect Dis..

[2] Bivona G, Agnello L, Ciaccio M (2021). Biomarkers for Prognosis and Treatment Response in COVID-19 Patients. Ann Lab Med..

[3] Malik P, Patel U, Mehta D, Patel N, Kelkar R, Akrmah M (2021). Biomarkers and outcomes of COVID-19 hospitalisations: systematic review and meta-analysis. BMJ Evid Based Med..

[4] Izcovich A, Ragusa MA, Tortosa F, Lavena Marzio MA, Agnoletti C, Bengolea A (2020). Prognostic factors for severity and mortality in patients infected with COVID-19: A systematic review. PLoS ONE..

[5] Wang D, Hu B, Hu C, Zhu F, Liu X, Zhang J (2020). Clinical Characteristics of 138 Hospitalized Patients With 2019 Novel Coronavirus-Infected Pneumonia in Wuhan. China. Jama..

[6] Gupta D, Jain A, Chauhan M, Dewan S (2022). Inflammatory Markers as Early Predictors of Disease Severity in COVID-19 Patients Admitted to Intensive Care Units: A Retrospective Observational Analysis. Indian J Crit Care Med..

[7] Xu Z, Zhang Y, Zhang C, Xiong F, Zhang J, Xiong J (2022). Clinical Features and Outcomes of COVID-19 Patients with Acute Kidney Injury and Acute Kidney Injury on Chronic Kidney Disease. Aging Dis..

[8] Petrilli CM, Jones SA, Yang J, Rajagopalan H, O’Donnell L, Chernyak Y (2020). Factors associated with hospital admission and critical illness among 5279 people with coronavirus disease 2019 in New York City: prospective cohort study. Bmj..

[9] Bowring MG, Wang Z, Xu Y, Betz J, Muschelli J, Garibaldi BT (2021). Outcome-Stratified Analysis of Biomarker Trajectories for Patients Infected With Severe Acute Respiratory Syndrome Coronavirus 2. Am J Epidemiol..

[10] Boss AN, Banerjee A, Mamalakis M, Ray S, Swift AJ, Wilkie C, et al. Development of a Mortality Prediction Model in Hospitalised SARS-CoV-2 Positive Patients Based on Routine Kidney Biomarkers. Int J Mol Sci. 2022;23(13). 10.3390/ijms23137260. 10.3390/ijms23137260 PMC9266863 35806273

[11] Syed AH, Khan T, Alromema N. A Hybrid Feature Selection Approach to Screen a Novel Set of Blood Biomarkers for Early COVID-19 Mortality Prediction. Diagnostics (Basel). 2022;12(7). 10.3390/diagnostics12071604. 10.3390/diagnostics12071604 PMC9316550 35885508

[12] Haitao T, Vermunt JV, Abeykoon J, Ghamrawi R, Gunaratne M, Jayachandran M (2020). COVID-19 and Sex Differences: Mechanisms and Biomarkers. Mayo Clin Proc..

[13] Lumish HS, Kim E, Selvaggi C, Cao T, Gupta A, Foulkes AS (2022). Biomarkers of Cardiac Injury, Renal Injury, and Inflammation Are Strong Mediators of Sex-Associated Death in COVID-19. Front Cardiovasc Med..

[14] Megiorni F, Pontecorvi P, Gerini G, Anastasiadou E, Marchese C, Ceccarelli S. Sex-Related Factors in Cardiovascular Complications Associated to COVID-19. Biomolecules. 2021;12(1). 10.3390/biom12010021. 10.3390/biom12010021 PMC8773922 35053169

[15] Lin H, McCulloch CE, Mayne ST (2002). Maximum likelihood estimation in the joint analysis of time-to-event and multiple longitudinal variables. Stat Med..

[16] Hickey G, Philipson P, Jorgensen A, Kolamunnage-Donà R. JoineRML: A joint model and software package for time-to-event and multivariate longitudinal outcomes. BMC Med Res Methodol. 2018;18. 10.1186/s12874-018-0502-1. 10.1186/s12874-018-0502-1 PMC6047371 29879902

[17] Rizopoulos D, Ghosh P (2011). A Bayesian semiparametric multivariate joint model for multiple longitudinal outcomes and a time-to-event. Stat Med..

[18] Chen Y, Wang Y (2017). Variable selection for joint models of multivariate longitudinal measurements and event time data. Stat Med..

[19] He Z, Tu W, Wang S, Fu H, Yu Z (2015). Simultaneous variable selection for joint models of longitudinal and survival outcomes. Biometrics..

[20] Wang JL, Chiou JM, Müller HG (2016). Functional Data Analysis. Ann Rev Stat Appl..

[21] Li K, Luo S (2019). Dynamic prediction of Alzheimer’s disease progression using features of multiple longitudinal outcomes and time-to-event data. Stat Med..

[22] Lin J, Li K, Luo S (2021). Functional survival forests for multivariate longitudinal outcomes: Dynamic prediction of Alzheimer’s disease progression. Stat Methods Med Res..

[23] Jiang BS, Xie Y, Colditz GA. Functional Ensemble Survival Tree: Dynamic Prediction of Alzheimer’s Disease Progression Accommodating Multiple Time-Varying Covariates. bioRxiv. 2020. 10.1101/2020.02.17.952994.

[24] Simon N, Friedman J, Hastie T, Tibshirani R (2013). A Sparse-Group Lasso. J Comput Graph Stat..

[25] Yao F, Müller HG, Wang JL (2005). Functional Data Analysis for Sparse Longitudinal Data. J Am Stat Assoc..

[26] Gajardo A, Bhattacharjee S, Carroll C, Chen Y, Dai X, Fan J, et al. fdapace: Functional Data Analysis and Empirical Dynamics. 2021. R package version 0.5.8. https://CRAN.R-project.org/package=fdapace. Accessed Sept 2023.

[27] van Buuren S, Groothuis-Oudshoorn K (2011). mice: Multivariate Imputation by Chained Equations in R. J Stat Softw..

[28] Yan F, Lin X, Huang X (2017). Dynamic prediction of disease progression for leukemia patients by functional principal component analysis of longitudinal expression levels of an oncogene. Ann Appl Stat..

[29] Cheng Y, Luo R, Wang K, Zhang M, Wang Z, Dong L (2020). Kidney disease is associated with in-hospital death of patients with COVID-19. Kidney Int..

[30] Wang Y, Perlman S (2022). COVID-19: Inflammatory Profile. Annu Rev Med..

[31] Wang F, Hou H, Luo Y, Tang G, Wu S, Huang M, et al. The laboratory tests and host immunity of COVID-19 patients with different severity of illness. JCI Insight. 2020;5(10). 10.1172/jci.insight.137799. 10.1172/jci.insight.137799 PMC7259533 32324595

[32] Chalmers S, Khawaja A, Wieruszewski PM, Gajic O, Odeyemi Y (2019). Diagnosis and treatment of acute pulmonary inflammation in critically ill patients: The role of inflammatory biomarkers. World J Crit Care Med..

[33] Rizopoulos D. The R Package JMbayes for Fitting Joint Models for Longitudinal and Time-to-Event Data using MCMC. arXiv. 2014. 10.48550/ARXIV.1404.7625.

