# Supplementary material for: Functional principal component analysis and sparse-group LASSO to identify associations between biomarker trajectories and mortality among hospitalized SARS-CoV-2 infected individuals
Source: BMC Med Res Methodol. 2023 Oct 28;23:254. doi: 10.1186/s12874-023-02076-3 (PMC10613396; doi:10.1186/s12874-023-02076-3)
Supplement: Supplementary file 1 — Additional file 1. Supplementary Figures and Tables. [file 12874_2023_2076_MOESM1_ESM.pdf]

Supplementary Figure 1. The mean function and the first three eigenfunctions (FPCs) of the 20 biomarkers, among females and males respectively

Blood urea nitrogen, females

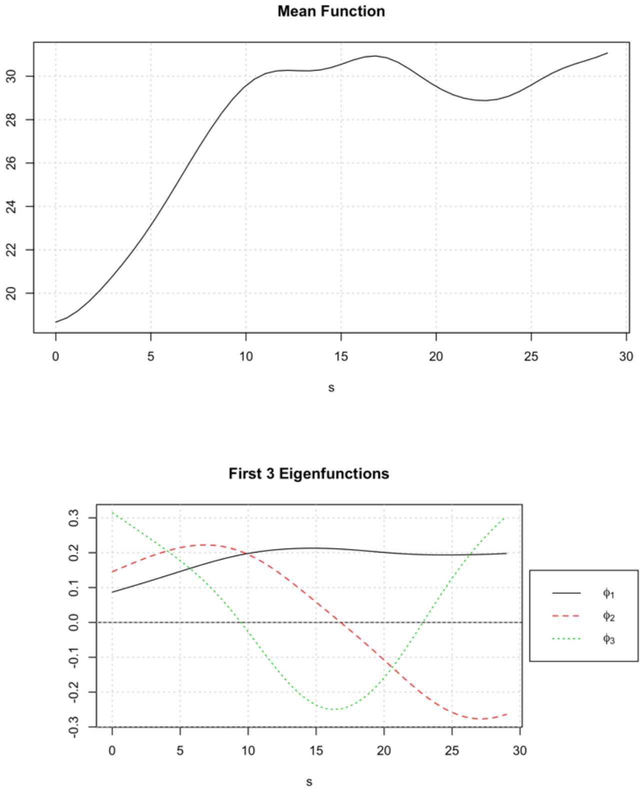

Blood urea nitrogen, males

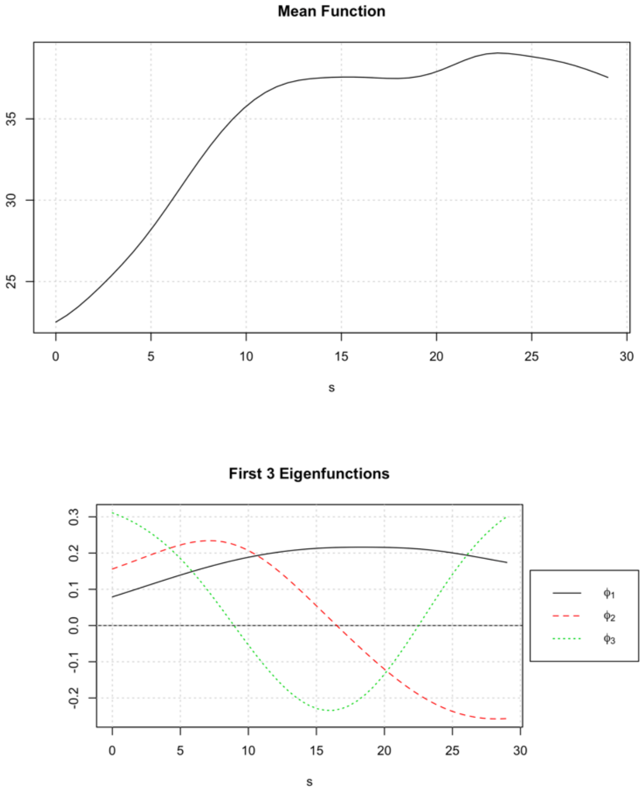

Creatinine, females

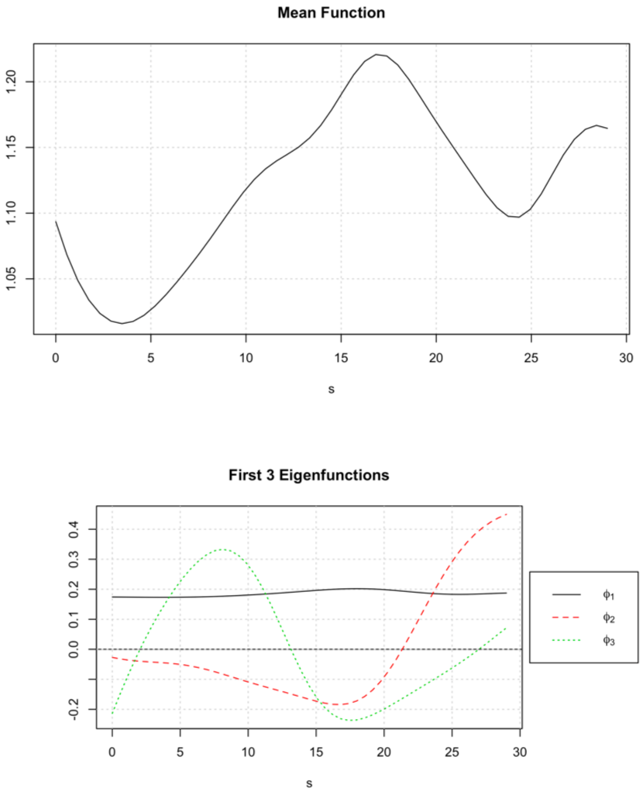

Creatinine, males

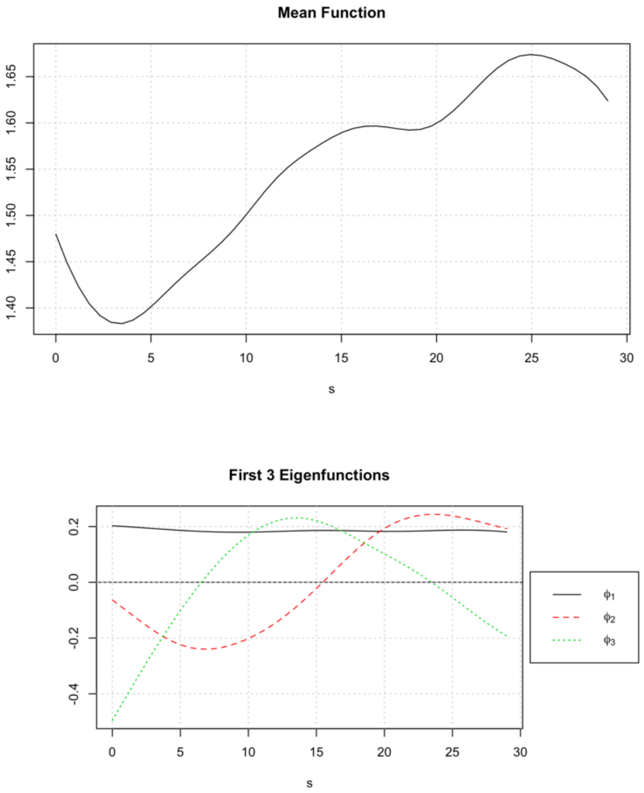

Estimated glomerular filtration rate, females

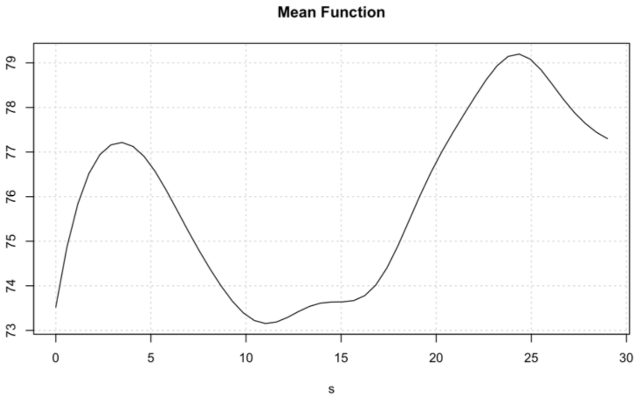

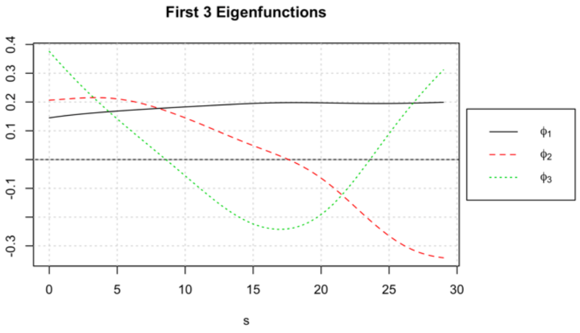

Estimated glomerular filtration rate, males

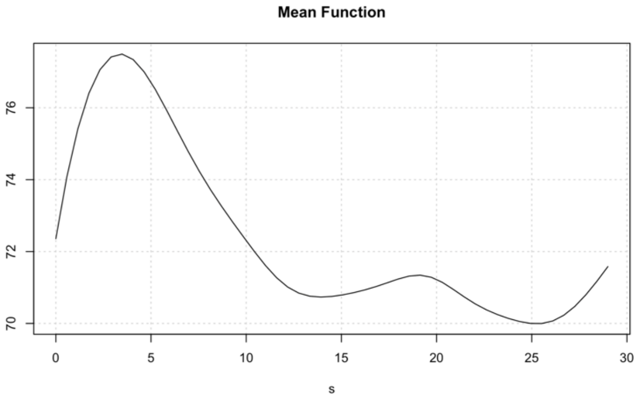

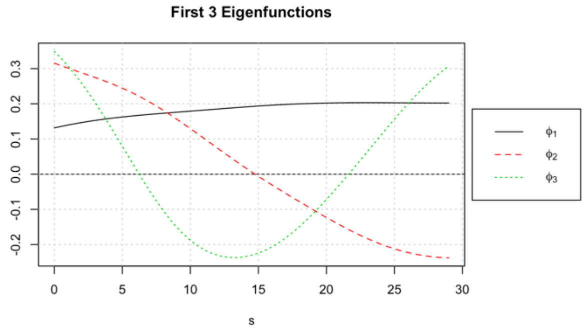

Creatine phosphokinase, females

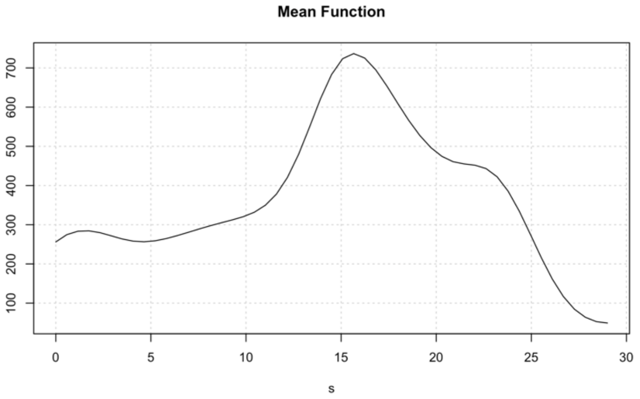

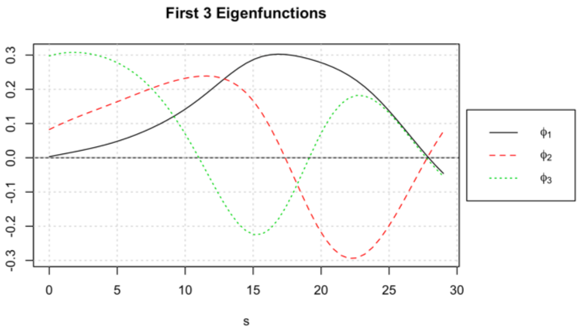

Creatine phosphokinase, males

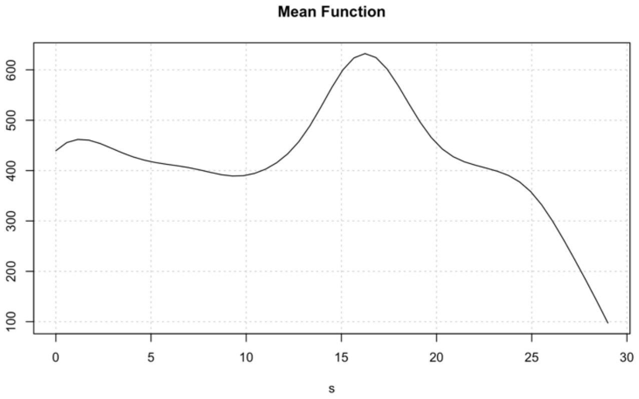

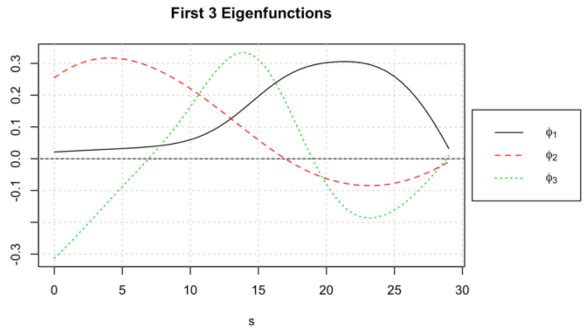

D-dimer, females

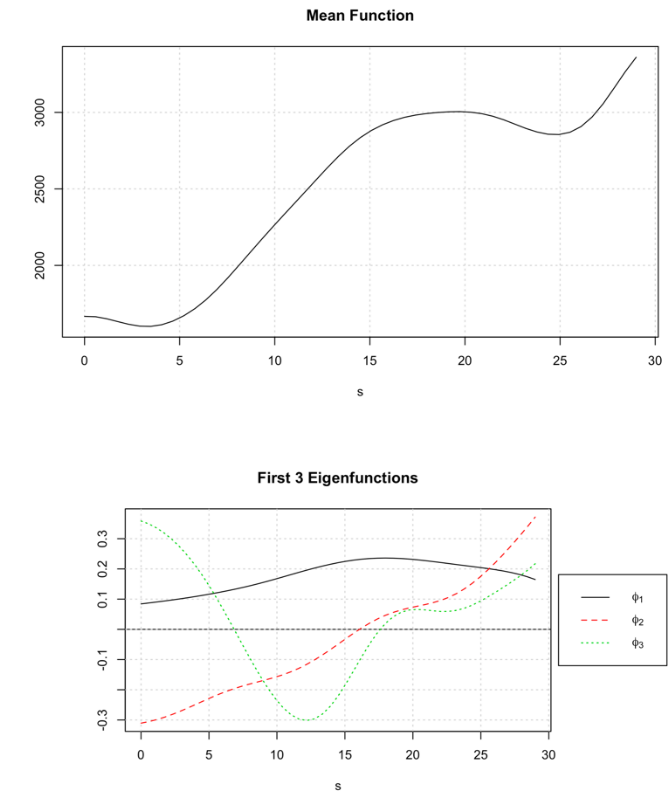

D-dimer, males

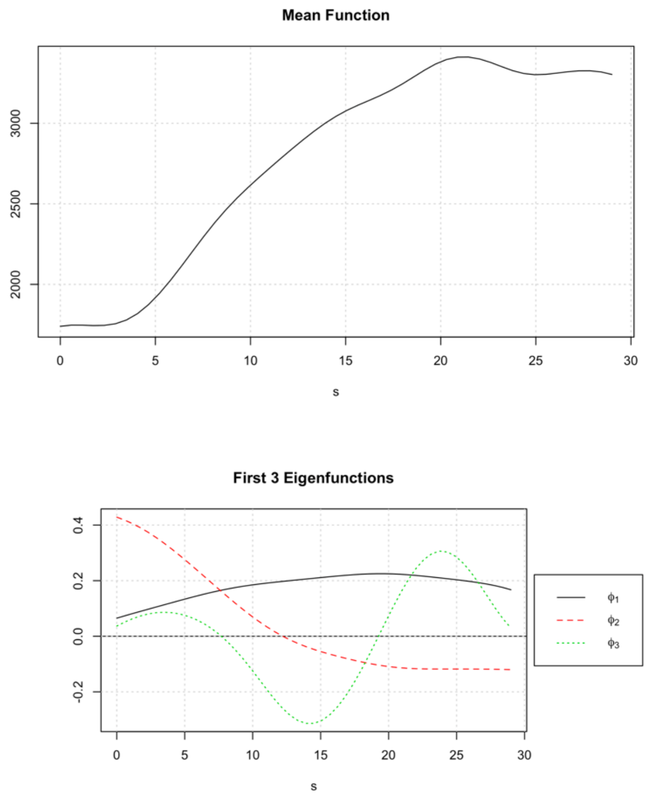

C-reactive protein, females

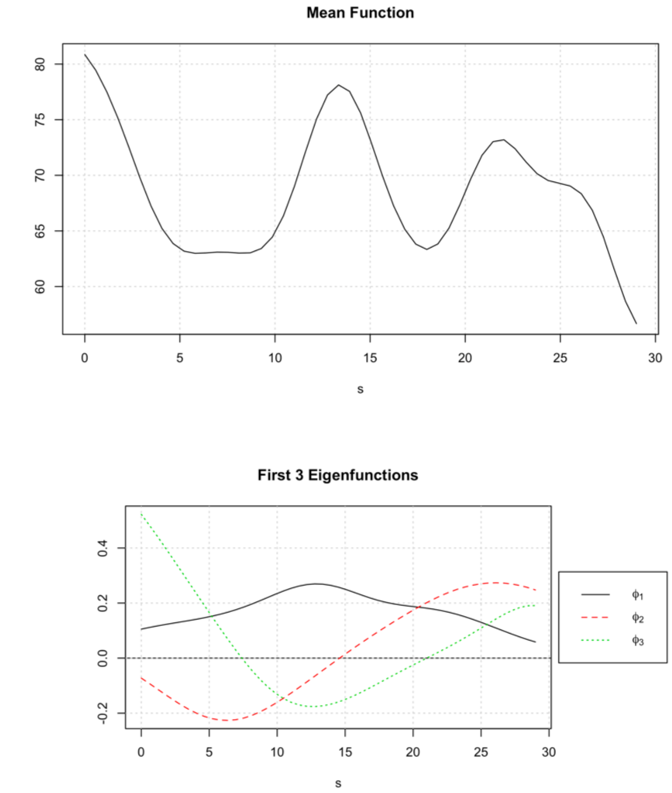

C-reactive protein, males

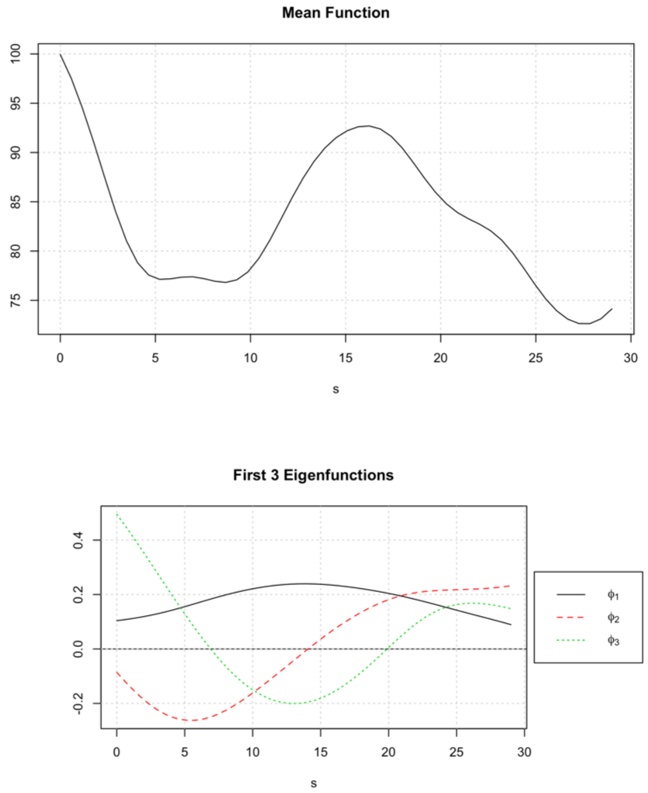

White blood cell count, females

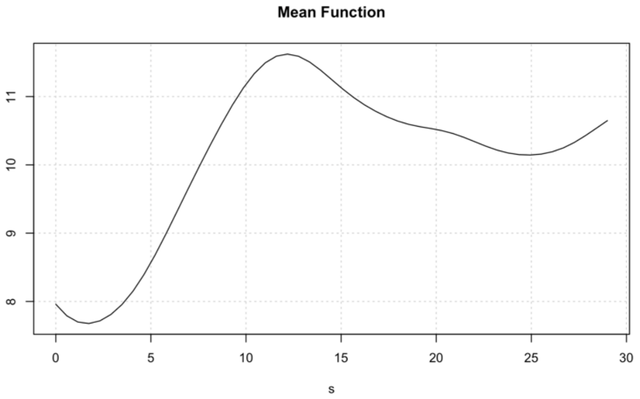

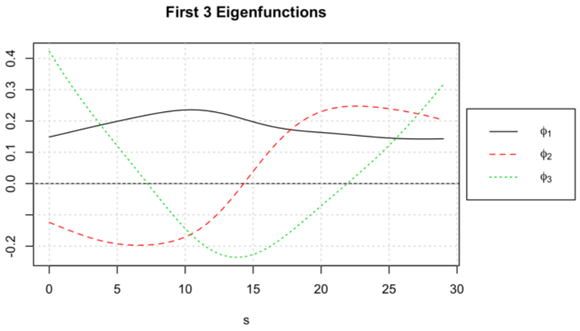

White blood cell count, males

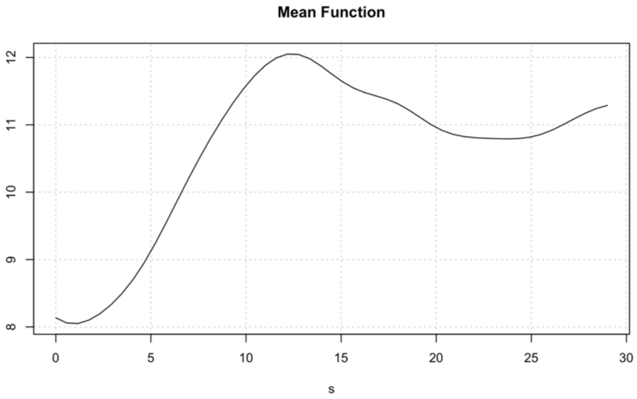

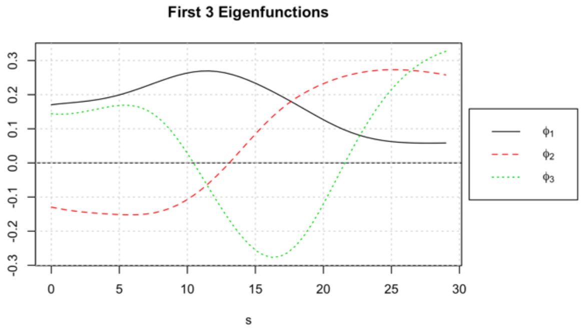

Absolute lymphocyte count, females

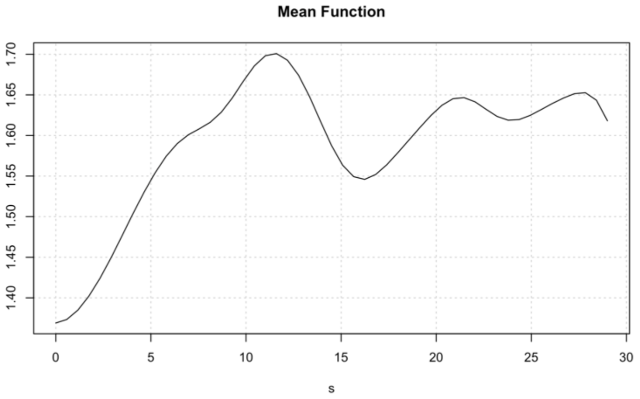

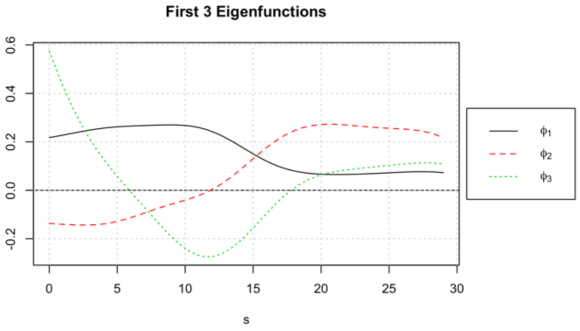

Absolute lymphocyte count, males

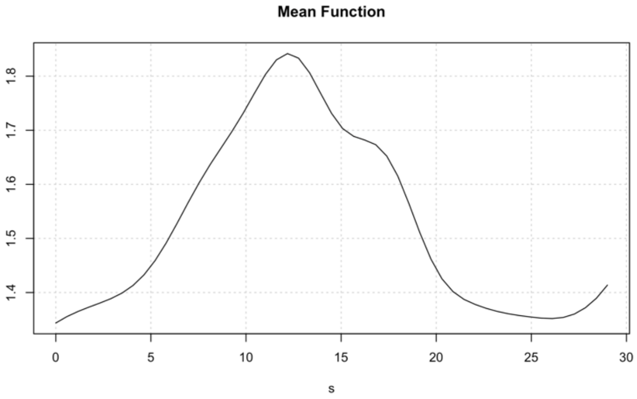

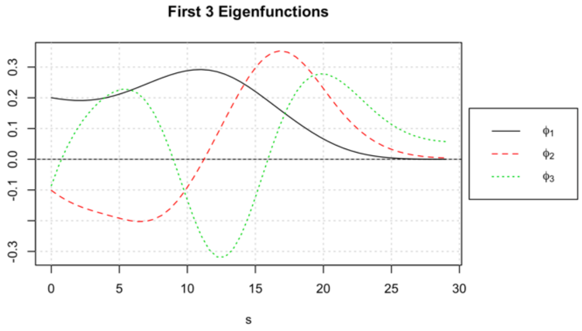

Ferritin, females

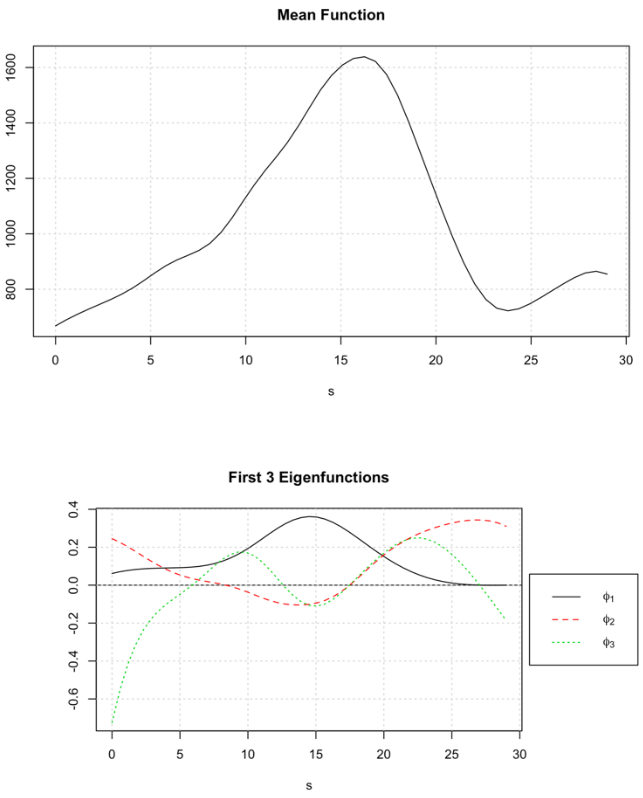

Ferritin, males

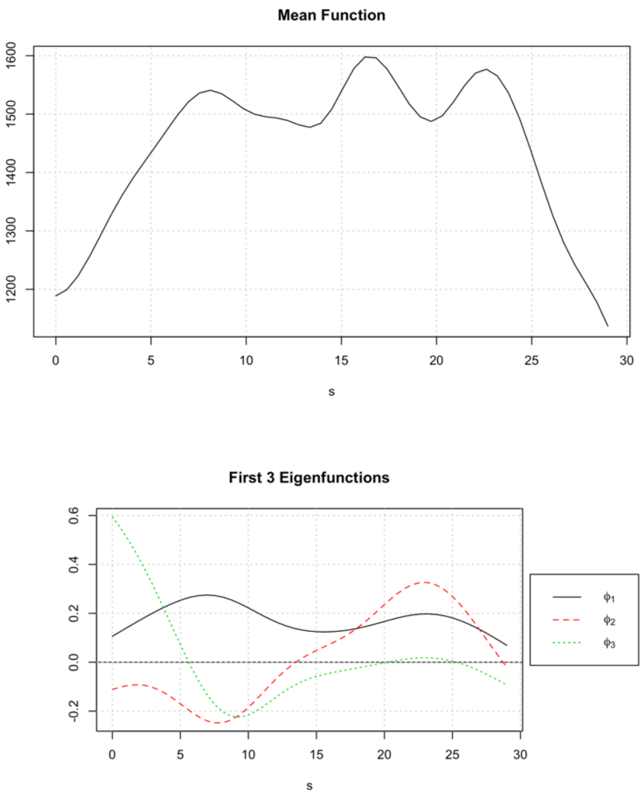

Hemoglobin, females

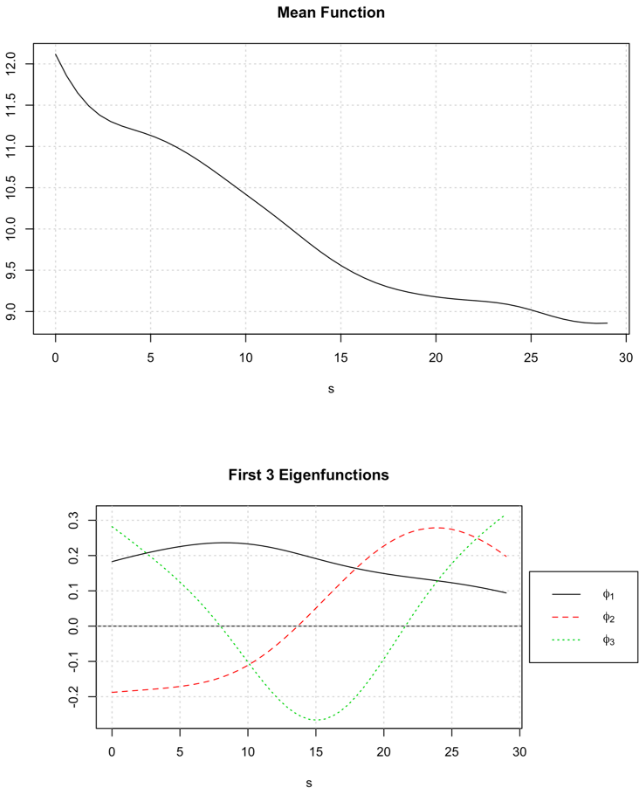

Hemoglobin, males

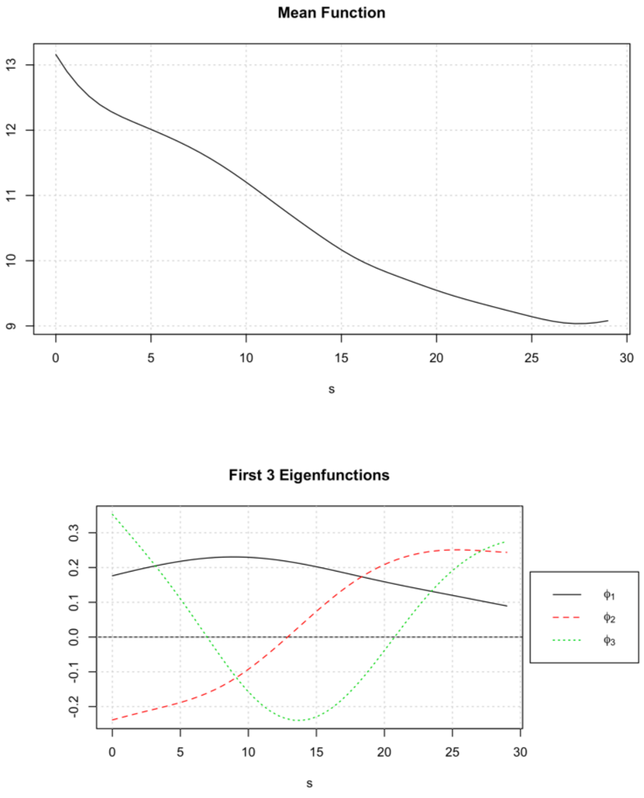

Hematocrit, females

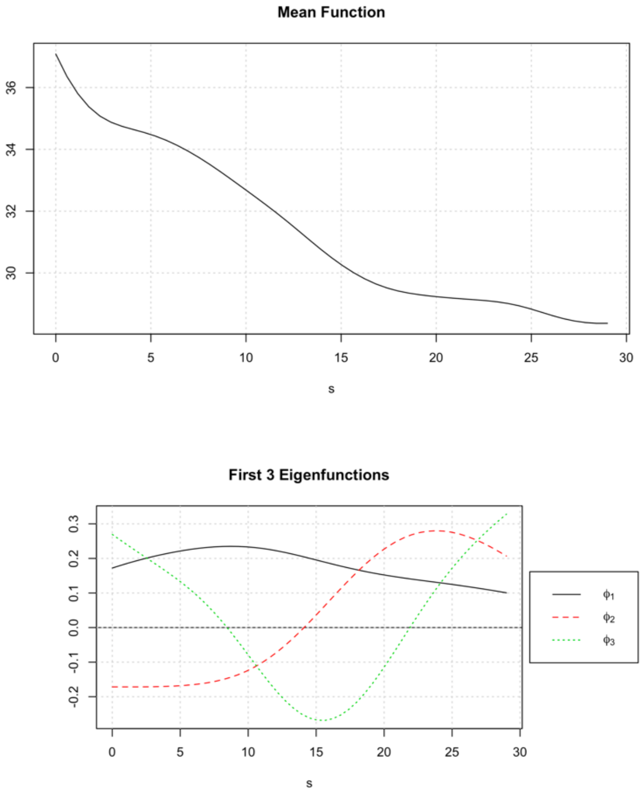

Hematocrit, males

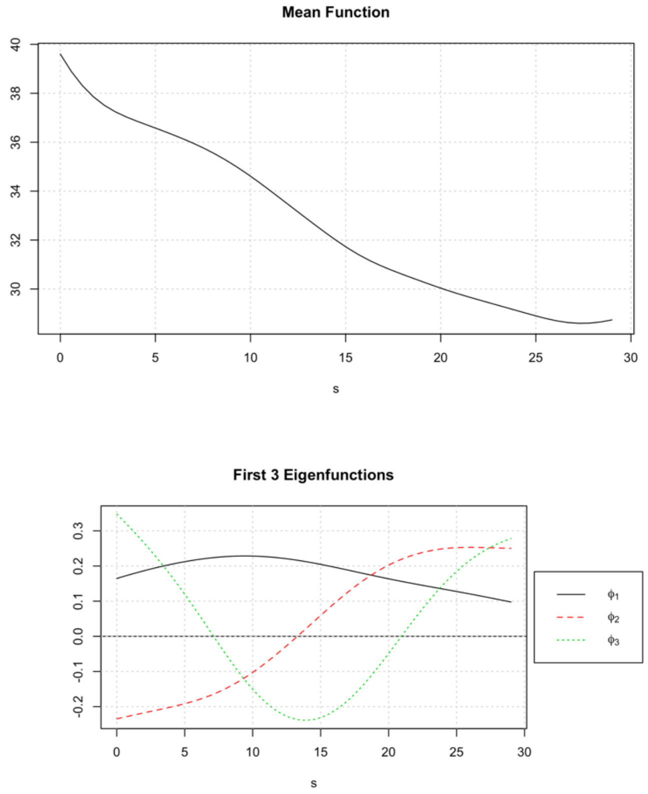

Platelets, females

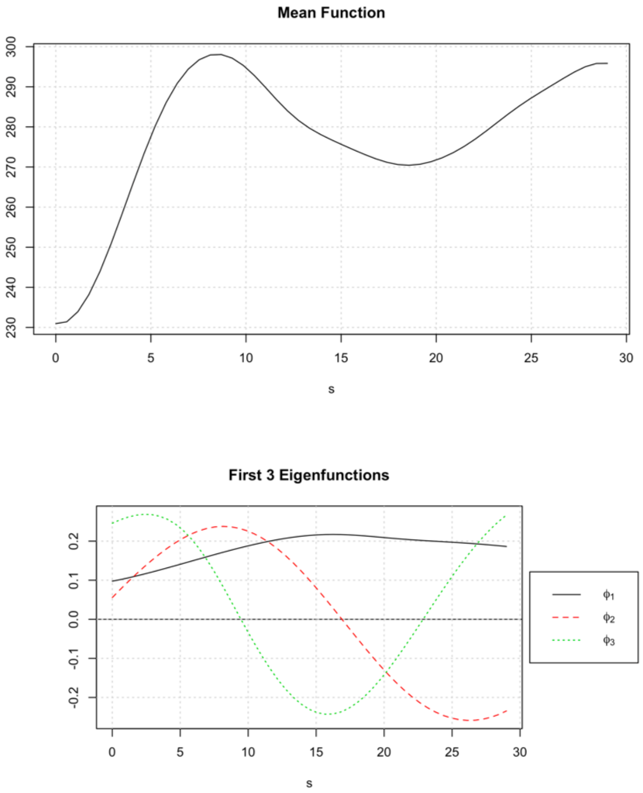

Platelets, males

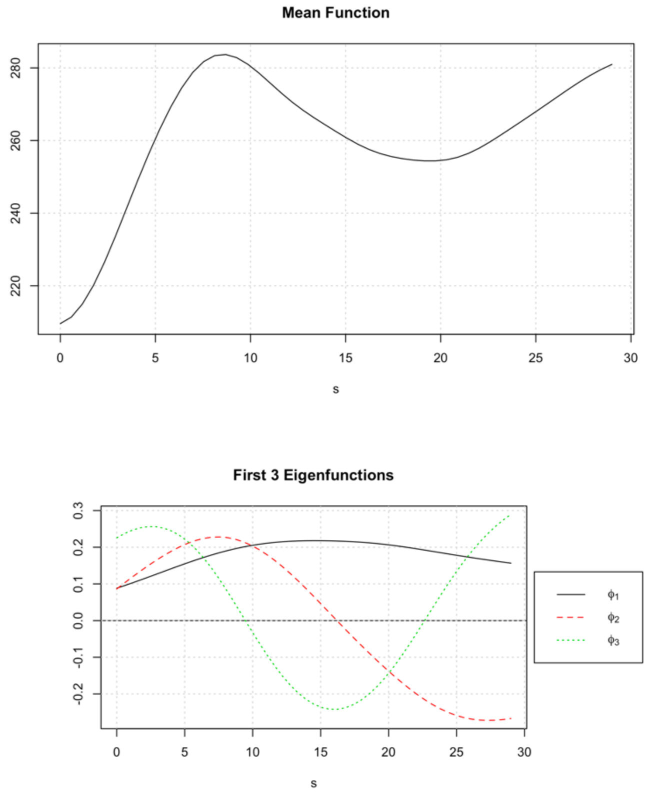

Alanine aminotransferase, females

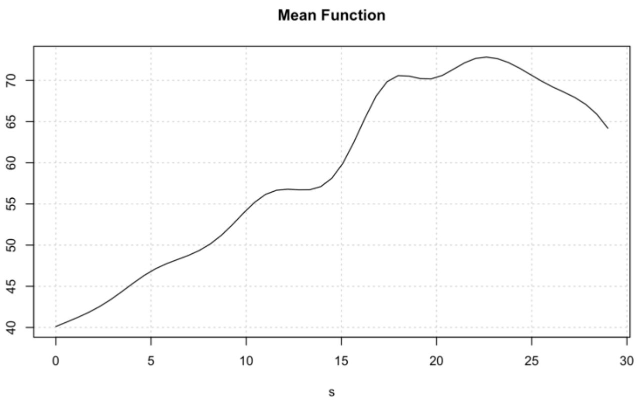

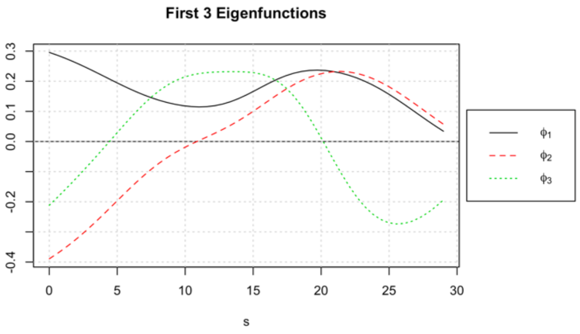

Alanine aminotransferase, males

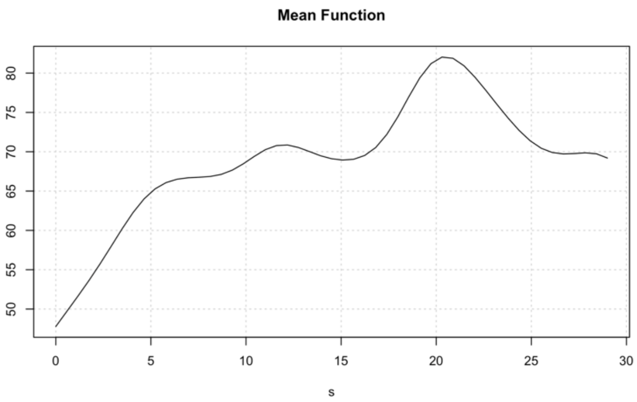

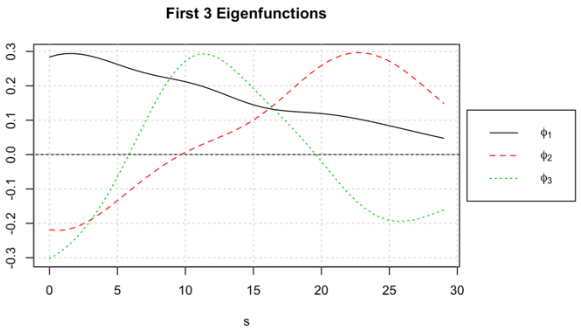

Aspartate aminotransferase, females

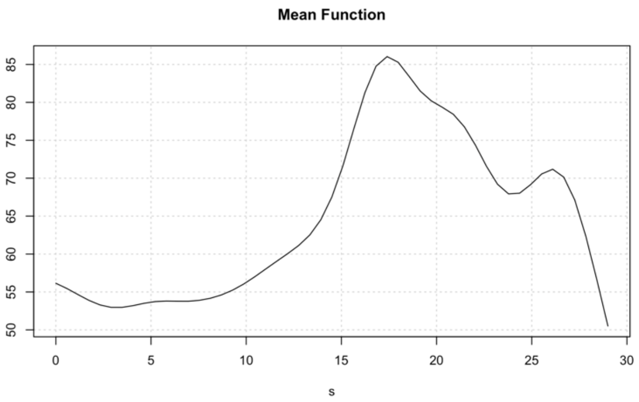

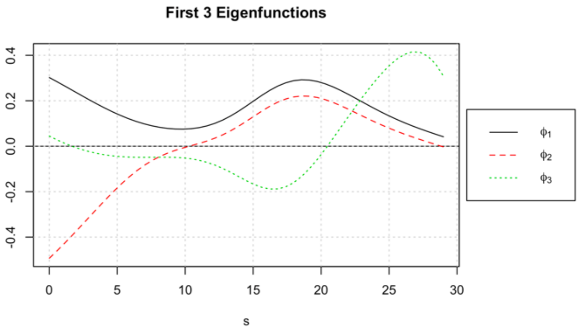

Aspartate aminotransferase, males

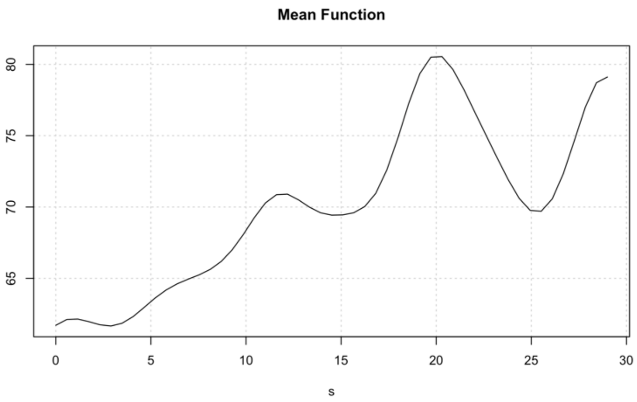

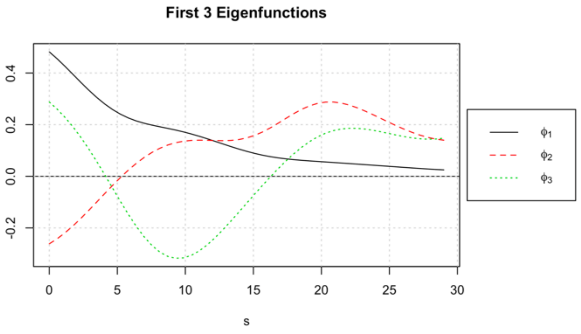

Alkaline phosphatase, females

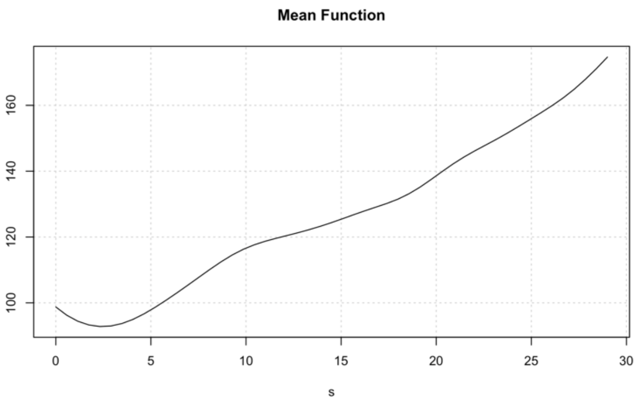

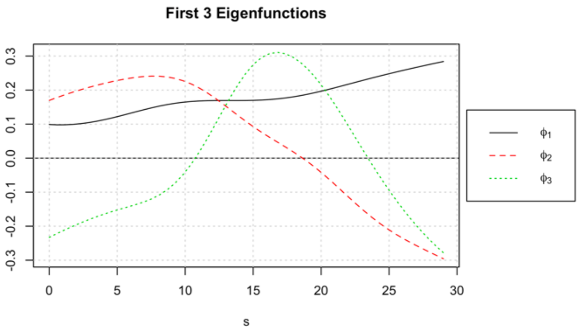

Alkaline phosphatase, males

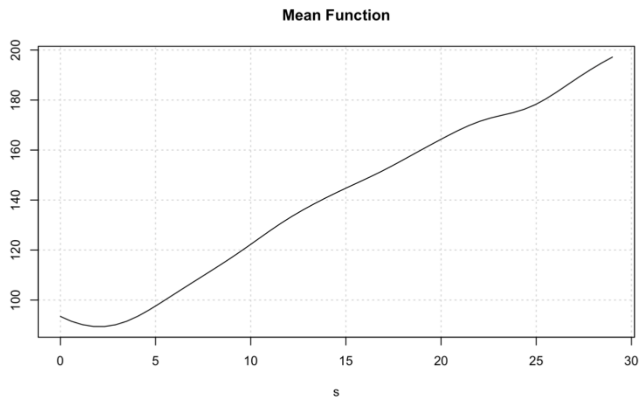

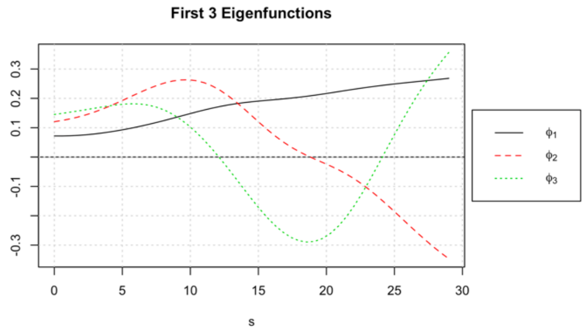

Total bilirubin, females

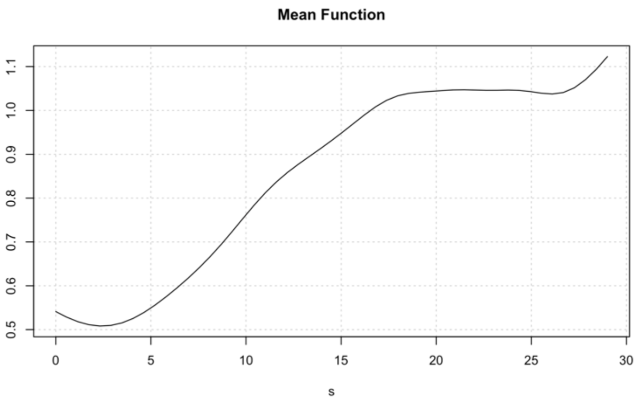

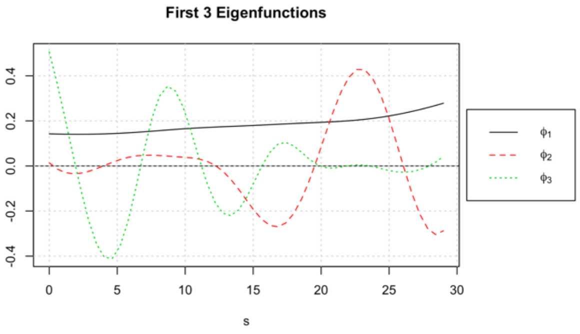

Total bilirubin, males

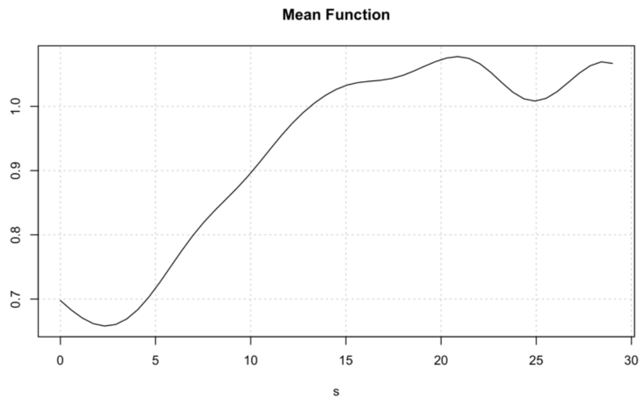

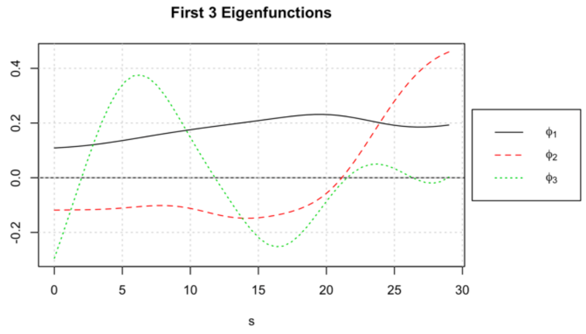

Albumin, females

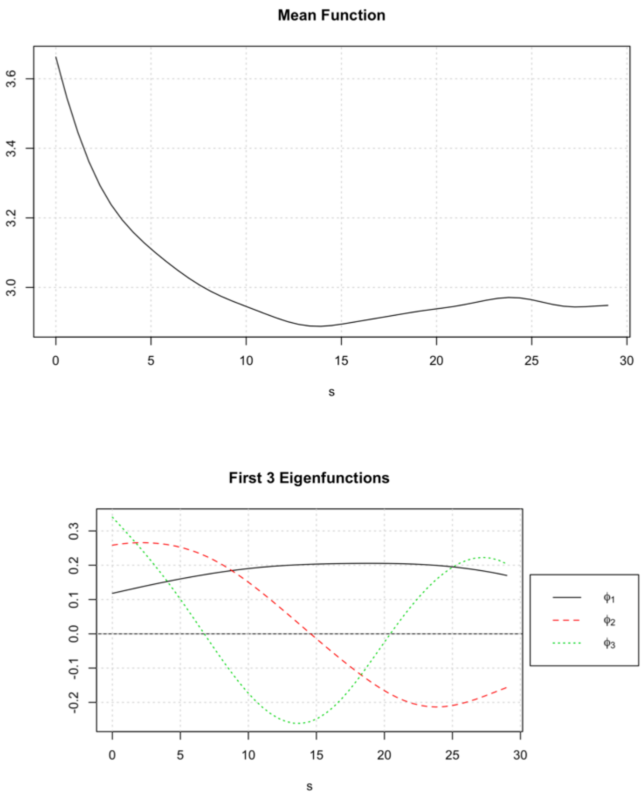

Albumin, males

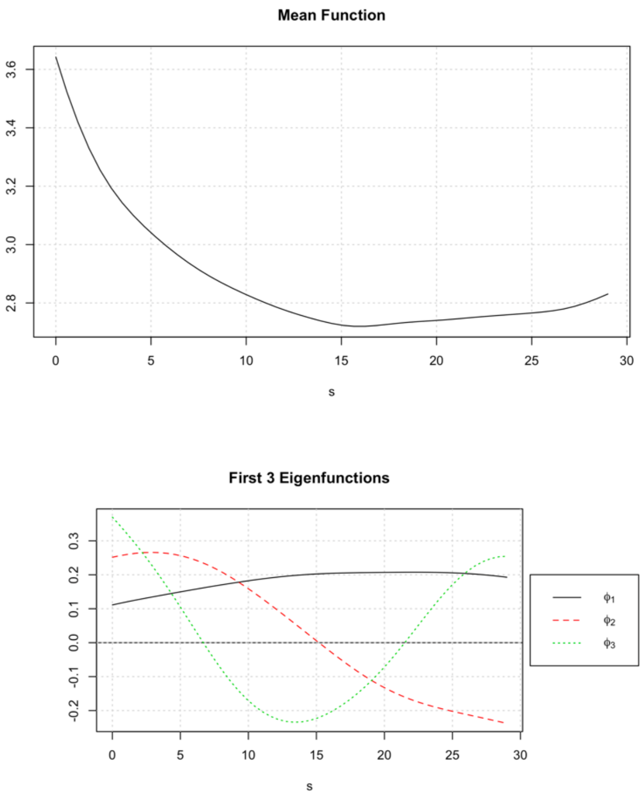

Lactate dehydrogenase, females

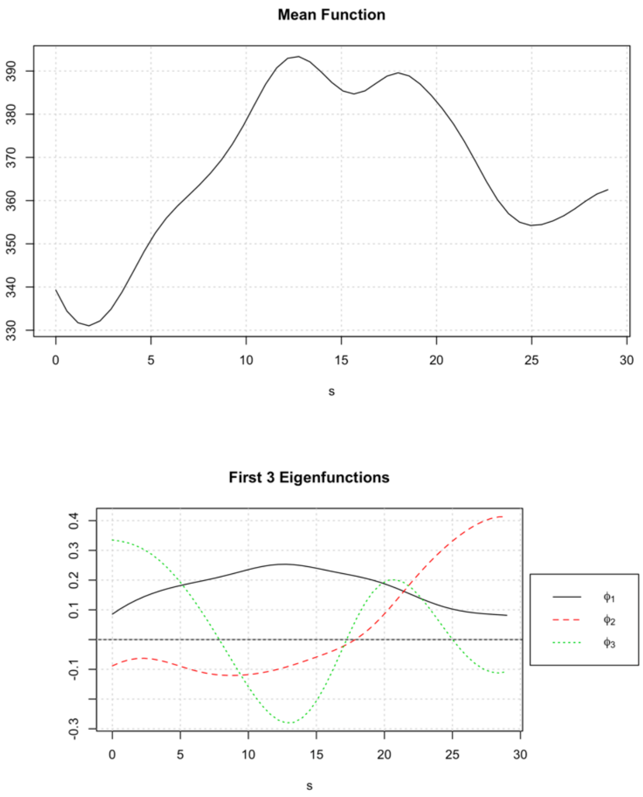

Lactate dehydrogenase, males

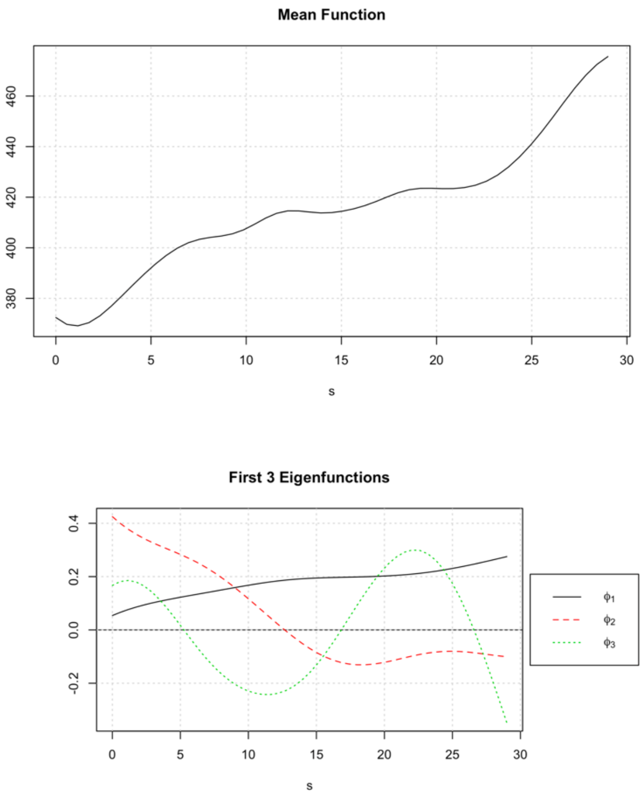

Glucose, females

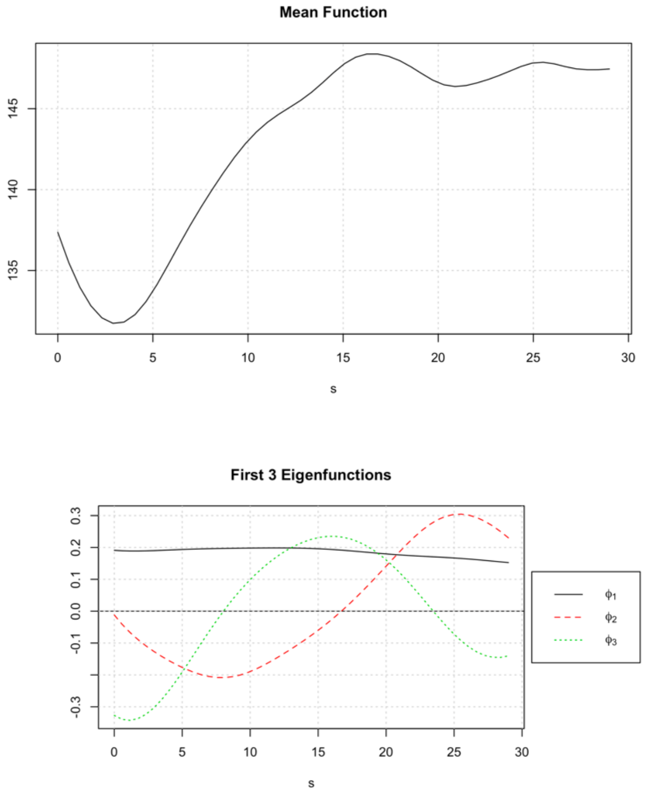

Glucose, males

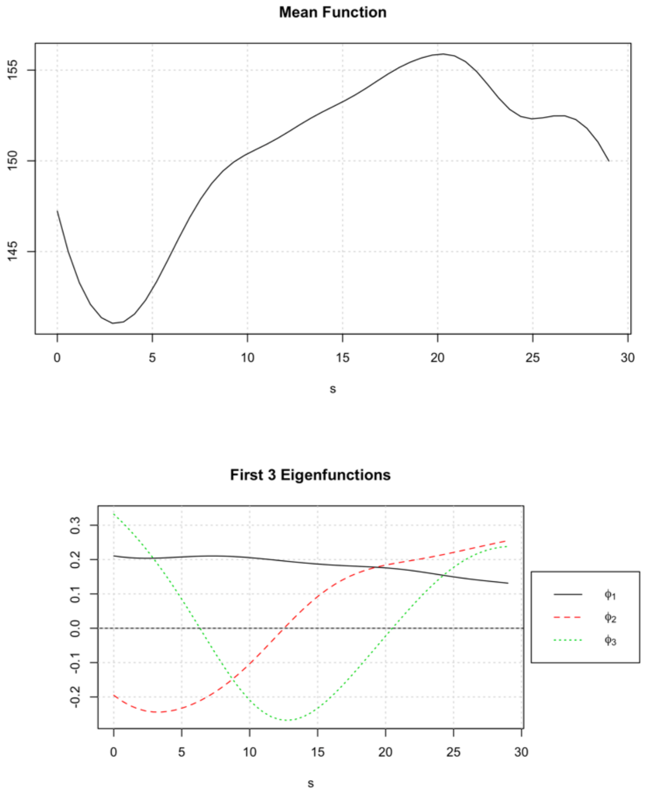

Anion gap, females

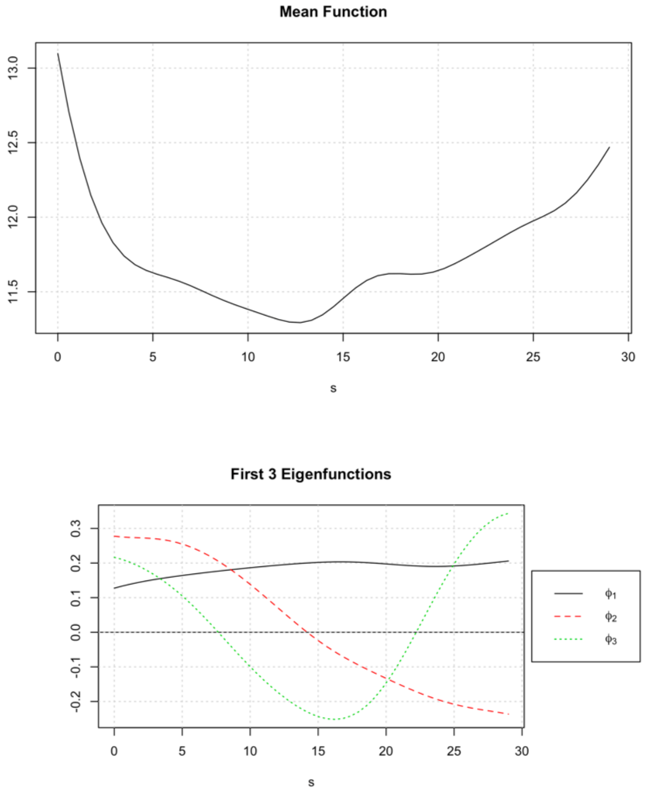

Anion gap, males

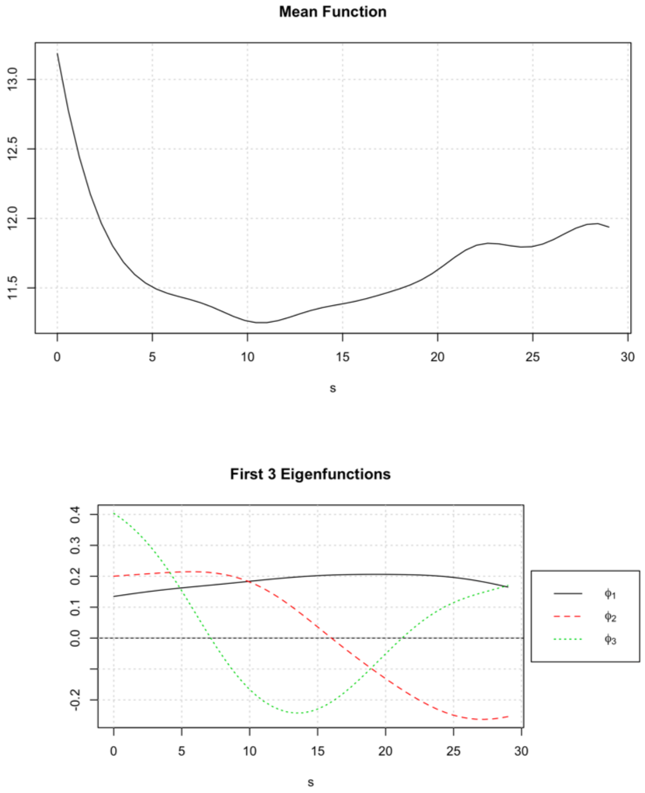

Supplementary Figure 2. Histogram of the first three FPC scores for the 20 biomarkers after imputation and standardization, among females (Panel A) and males (Panel B) respectively

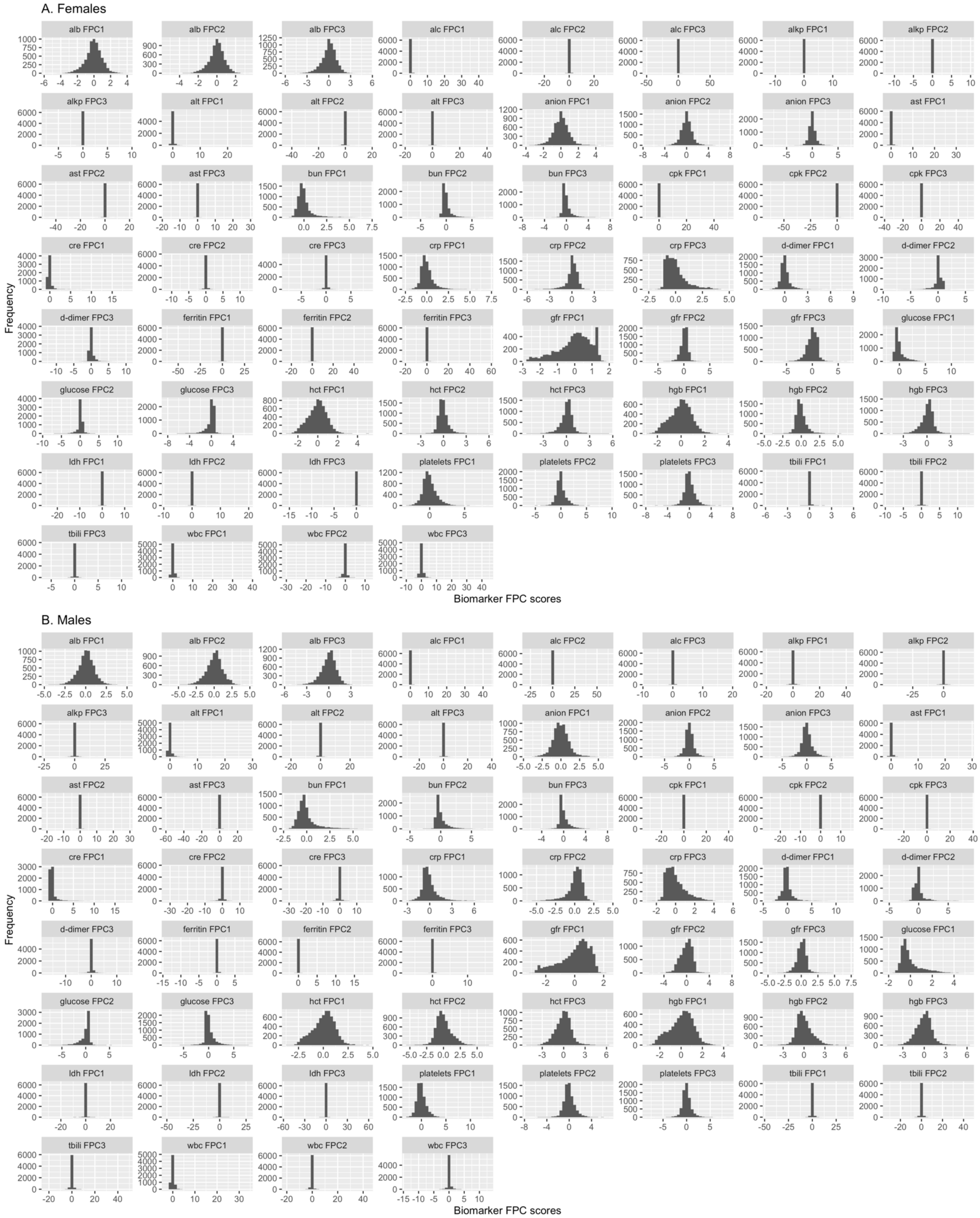

Supplementary Figure 3. Visualization example of how each patient's FPC scores could represent the variation of their individual biomarker trajectories from the mean function. The mean function (Panel a) and the first three eigenfunctions (Panel B) estimated from FPCA for blood urea nitrogen (bun) among males are the same as shown in Supplementary Figure 1. Panel C illustrates individual trajectories of bun of three male patients with different FPC scores (Panel D).

A.

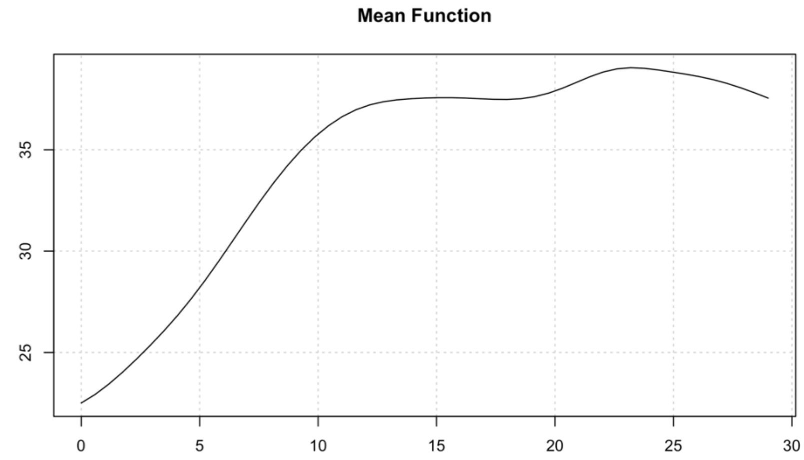

C.

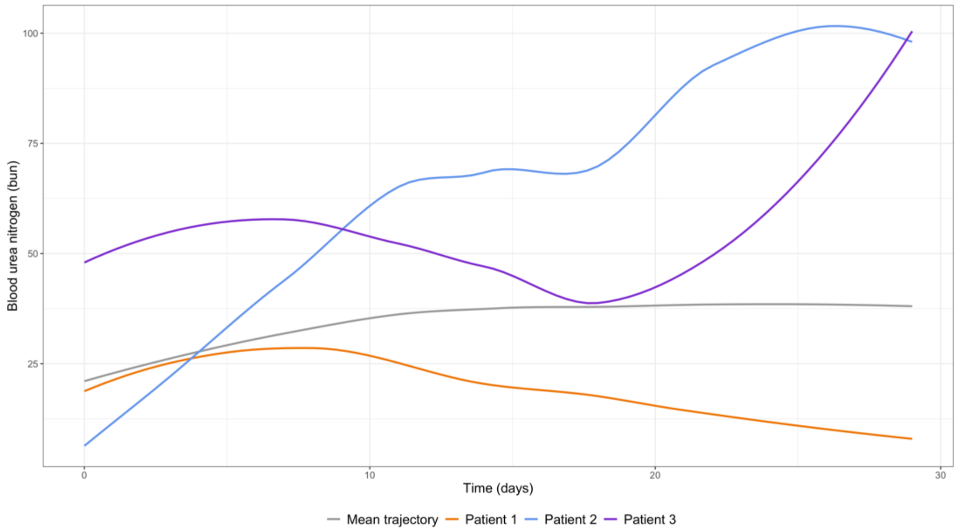

B.

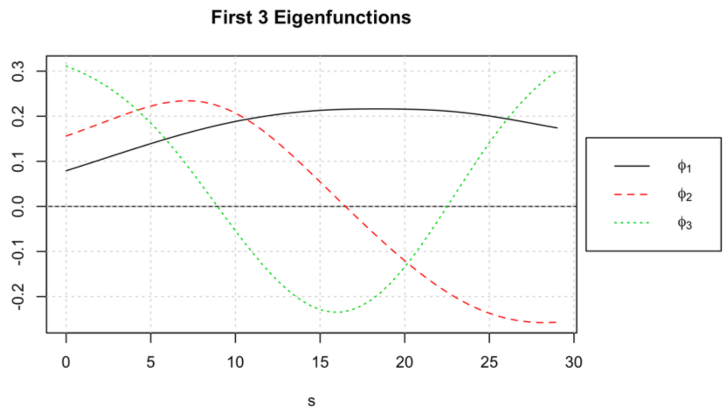

D.

	Scores for FPC1 (A_{1i1})	Scores for FPC2 (A_{1i2})	Scores for FPC3 (A_{1i3})
Patient 1	-88	44	-1
Patient 2	185	-103	2
Patient 3	102	2	81

Supplementary Figure 4. Histogram of the baseline (Panel A and B) and peak (Panel C and D) measurements for the 20 biomarkers after log transformation, imputation and standardization, among females (Panel A and C) and males (Panel B and D) respectively

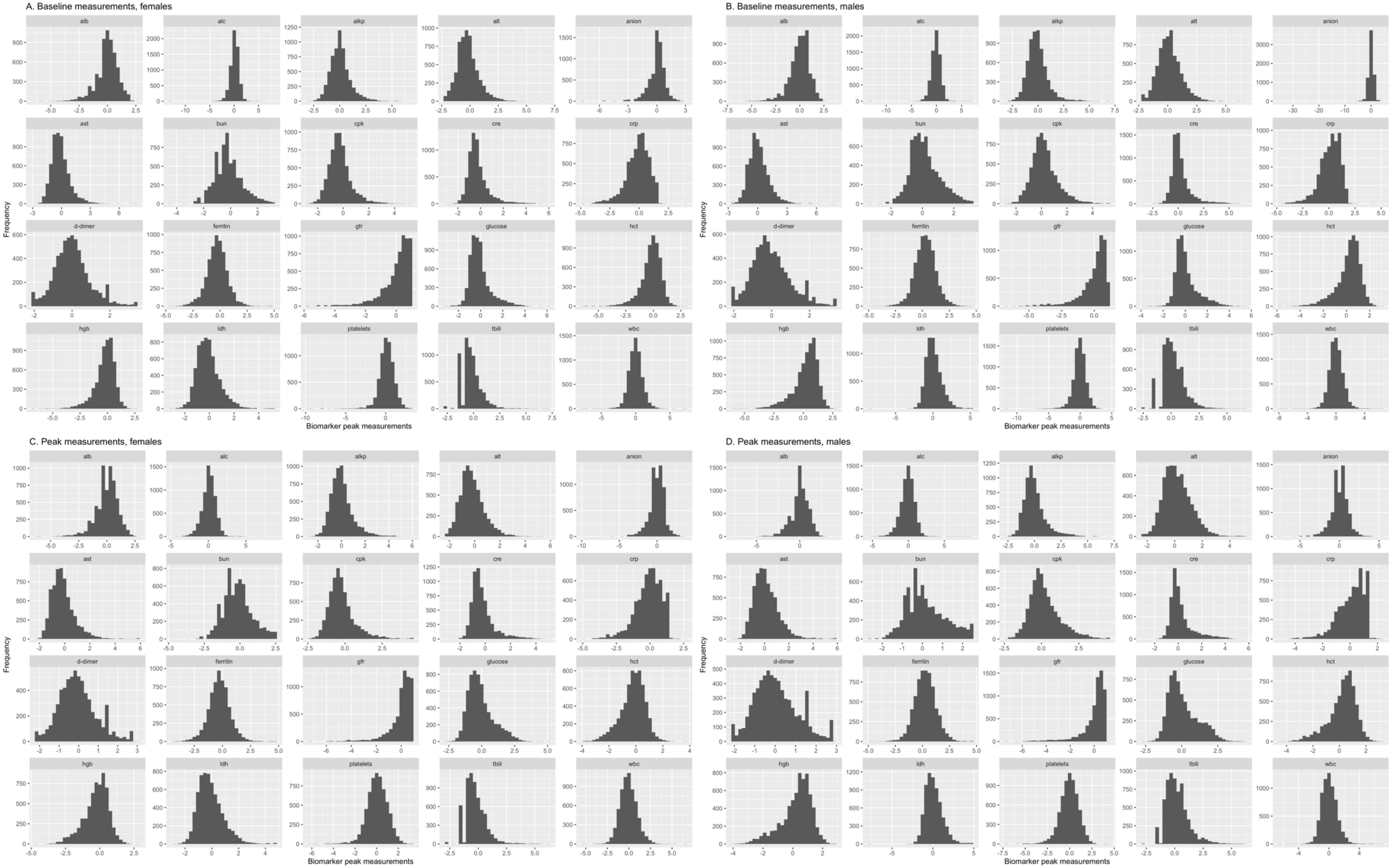

Supplementary Figure 5. Sensitivity analysis: Estimated regression coefficients $\widehat{\boldsymbol{\beta}}$ from SGL models fitted with the scores of the first 3 FPCs of each biomarker as exposure variables, and with different weight (larger η implies more LASSO than group LASSO structure), tiles with no border or annotated numbers indicate $\widehat{\boldsymbol{\beta}}$ being regularized to zero
(The full names of the abbreviated biomarkers are listed at the end of the manuscript.)

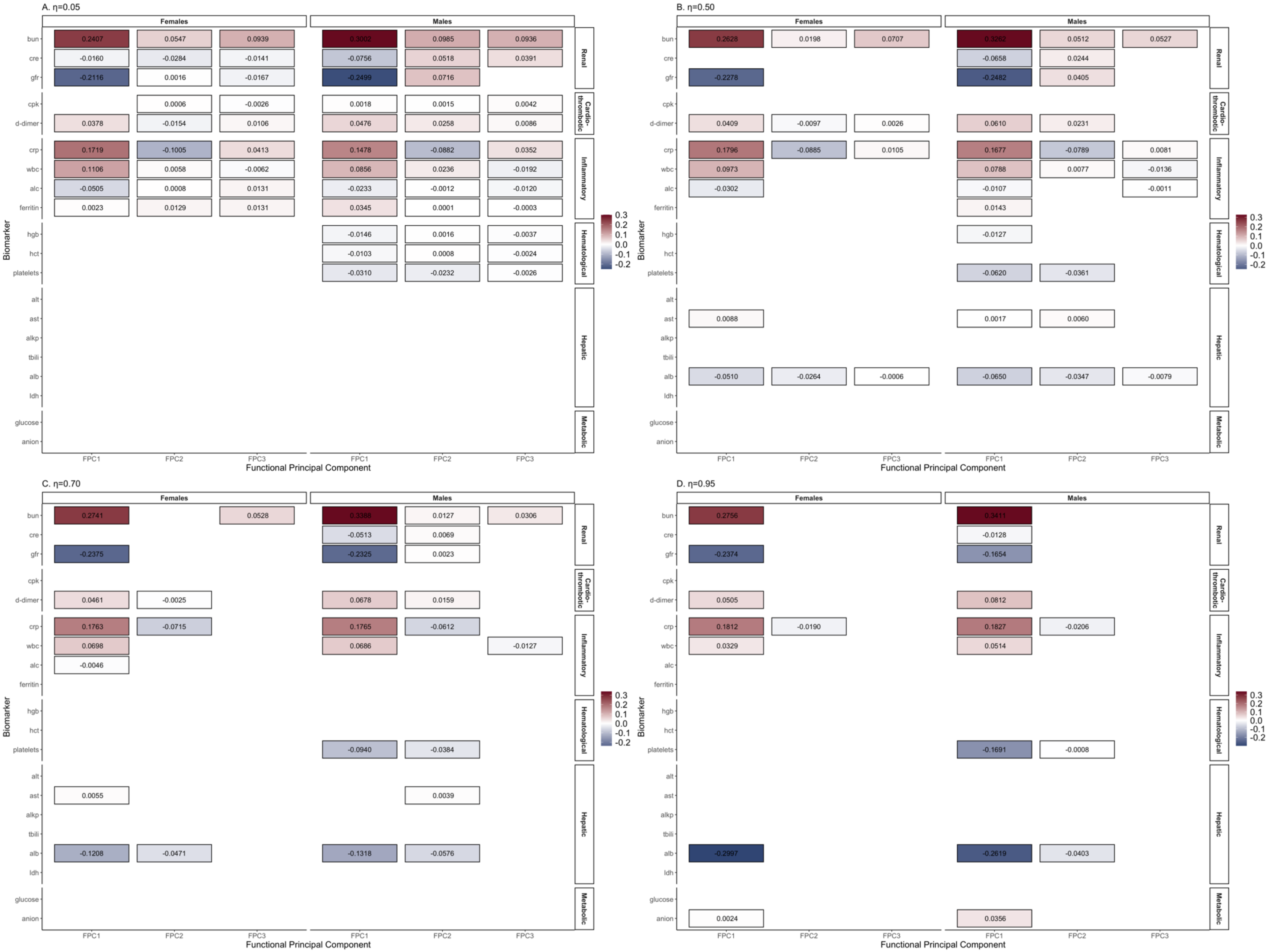

Supplementary Table 1. Summary statistics for the 20 biomarkers categorized into 6 groups based on their pathophysiological classifications

Biomarker groups	Biomarkers	Summary measures	Total	Female	Male
Renal	Blood urea nitrogen (bun)	Baseline	15 (11, 23.5)	14 (9, 21)	17 (12, 25.5)
		Peak	21 (14, 34)	19 (13, 30)	23 (16, 39)
		Number of subjects with ≥1 measurement	12589 (97.3%)	5960 (94.6%)	6629 (99.8%)
		Number of measurements	5 (3, 9)	5 (3, 8)	6 (3, 10)
	Creatinine (cre)	Baseline	0.95 (0.76, 1.25)	0.82 (0.68, 1.07)	1.05 (0.88, 1.375)
		Peak	1 (0.81, 1.38)	0.87 (0.71, 1.16)	1.11 (0.93, 1.55)
		Number of subjects with ≥1 measurement	12609 (97.4%)	5980 (94.9%)	6629 (99.8%)
		Number of measurements	5 (3, 9)	5 (3, 8)	6 (3, 10)
	Estimated glomerular filtration rate (gfr)	Baseline	77 (52, 97)	77 (52, 97)	77 (52, 96)
		Peak	93 (72, 108)	92 (72, 108)	94 (72, 108)
		Number of subjects with ≥1 measurement	12600 (97.4%)	5975 (94.8%)	6625 (99.8%)
		Number of measurements	5 (3, 9)	5 (3, 8)	6 (3, 10)
Cardio-thrombotic	Creatine phosphokinase (cpk)	Baseline	107 (59, 231)	82 (49, 161)	139 (73, 311.75)
		Peak	128 (67, 320)	94 (55, 205)	174 (86, 430)
		Number of subjects with ≥1 measurement	9183 (71.0%)	4237 (67.3%)	4946 (74.5%)
		Number of measurements	1 (0, 4)	1 (0, 3)	2 (0, 4)
	D-dimer	Baseline	1071 (631, 2035)	1080 (638, 1951)	1056 (623.875, 2104.75)
		Peak	1351 (729.5, 3095.5)	1279 (714, 2794)	1418.5 (738, 3462.25)
		Number of subjects with ≥1 measurement	6139 (47.4%)	2825 (44.8%)	3314 (49.9%)
		Number of measurements	0 (0, 3)	0 (0, 2)	0 (0, 3)
Inflammatory	C-reactive protein (crp)	Baseline	63.55 (22.3, 127)	53.7 (17.1, 108.8)	72.7 (29.15, 137.75)
		Peak	83.25 (33.9, 154.3)	69.9 (26.9, 141.5)	99.5 (42.3, 168.85)
		Number of subjects with ≥1 measurement	9404 (72.7%)	4357 (69.2%)	5047 (76.0%)
		Number of measurements	2 (0, 4)	2 (0, 4)	2 (1, 5)
	White blood cell count (wbc)	Baseline	7.03 (5.17, 9.59)	7.03 (5.08, 9.54)	7.03 (5.21, 9.63)
		Peak	9.15 (6.6, 13.13)	8.95 (6.47, 12.67)	9.38 (6.71, 13.525)
		Number of subjects with ≥1 measurement	12893 (99.6%)	6279 (99.7%)	6614 (99.6%)
		Number of measurements	5 (3, 9)	5 (2.75, 8)	5 (3, 10)
	Absolute lymphocyte count (alc)	Baseline	1.03 (0.69, 1.535)	1.12 (0.74, 1.63)	0.96 (0.65, 1.44)
		Peak	1.57 (1.09, 2.18)	1.66 (1.17, 2.28)	1.49 (1.03, 2.08)
		Number of subjects with ≥1 measurement	12384 (95.7%)	5822 (92.4%)	6562 (98.8%)
		Number of measurements	4 (2, 8)	4 (2, 7)	5 (2, 8)
	Ferritin	Baseline	488 (223, 978)	342.3 (160, 675)	656 (321.25, 1260)
		Peak	605.5 (278.5, 1244)	415 (194, 839)	837 (397, 1601.5)
		Number of subjects with ≥1 measurement	9319 (72.0%)	4340 (68.9%)	4979 (75.0%)
		Number of measurements	1 (0, 3)	1 (0, 3)	2 (0, 4)
Hematological	Hemoglobin (hgb)	Baseline	12.9 (11.5, 14.2)	12.4 (11.2, 13.5)	13.6 (12, 14.8)
		Peak	13.2 (11.8, 14.5)	12.6 (11.4, 13.7)	13.9 (12.35, 15.1)
		Number of subjects with ≥1 measurement	12893 (99.6%)	6279 (99.7%)	6614 (99.6%)
		Number of measurements	5 (3, 9)	5 (2, 8)	5 (3, 10)
	Hematocrit (hct)	Baseline	39.1 (35.3, 42.7)	37.8 (34.4, 40.9)	40.8 (36.5, 44.2)
		Peak	40.1 (36.3, 43.7)	38.6 (35.4, 41.7)	41.9 (37.7, 45.2)
		Number of subjects with ≥1 measurement	12893 (99.6%)	6279 (99.7%)	6614 (99.6%)
		Number of measurements	5 (3, 9)	5 (2, 8)	5 (3, 10)
	Platelets	Baseline	210 (160, 270)	220 (171, 282)	199 (153, 258)
		Peak	275 (206, 368)	279 (213, 370)	271 (200, 365)
		Number of subjects with ≥1 measurement	12887 (99.6%)	6276 (99.6%)	6611 (99.5%)
		Number of measurements	5 (3, 9)	5 (2, 8)	5 (3, 10)
Hepatic	Alanine aminotransferase (alt)	Baseline	25 (16, 43)	22 (14, 36)	29 (19, 50)
		Peak	34 (20, 69)	28 (17, 54)	42 (24, 83)
		Number of subjects with ≥1 measurement	12130 (93.7%)	5731 (91.0%)	6399 (96.4%)
		Number of measurements	4 (2, 7)	3 (1, 6)	4 (2, 7)
	Aspartate aminotransferase (ast)	Baseline	34 (23, 54)	31 (21.5, 48)	37 (25, 58.125)
		Peak	42 (27, 77)	37 (24, 64)	48 (29, 87.25)
		Number of subjects with ≥1 measurement	12131 (93.7%)	5731 (91.0%)	6400 (96.4%)
		Number of measurements	4 (2, 7)	3 (1, 6)	4 (2, 7)
	Alkaline phosphatase (alkp)	Baseline	79 (63, 104)	82 (64, 108)	78 (61, 101)
		Peak	87 (68, 119)	88 (68, 121)	86 (67, 118)
		Number of subjects with ≥1 measurement	12125 (93.7%)	5724 (90.9%)	6401 (96.4%)
		Number of measurements	4 (2, 7)	3 (1, 6)	4 (2, 7)
		Baseline	0.5 (0.3, 0.7)	0.4 (0.3, 0.6)	0.5 (0.4, 0.7)

	Total bilirubin (tbili)	Peak	0.5 (0.4, 0.8)	0.5 (0.3, 0.7)	0.6 (0.4, 0.9)
		Number of subjects with ≥1 measurement	12127 (93.7%)	5724 (90.9%)	6403 (96.4%)
		Number of measurements	4 (2, 7)	3 (1, 6)	4 (2, 7)
	Albumin (alb)	Baseline	3.7 (3.35, 4.05)	3.7 (3.4, 4)	3.7 (3.3, 4.05)
		Peak	3.8 (3.4, 4.1)	3.8 (3.4, 4.1)	3.8 (3.4, 4.1)
		Number of subjects with ≥1 measurement	12145 (93.8%)	5731 (91.0%)	6414 (96.6%)
		Number of measurements	4 (2, 7)	3 (1, 6)	4 (2, 8)
	Lactate dehydrogenase (ldh)	Baseline	301 (230, 406)	289 (224, 389)	313.5 (237, 424)
		Peak	334 (248, 464)	316.25 (240, 437)	350 (259, 487.25)
		Number of subjects with ≥1 measurement	9258 (71.5%)	4314 (68.5%)	4944 (74.4%)
		Number of measurements	1 (0, 3)	1 (0, 3)	2 (0, 4)
Metabolic	Glucose (glucose)	Baseline	119 (102, 150)	115 (99, 145)	121 (104, 155)
		Peak	143 (117, 202)	138.58 (113, 192)	148 (120, 212.08)
		Number of subjects with ≥1 measurement	12586 (97.3%)	5958 (94.6%)	6628 (99.8%)
		Number of measurements	5 (3, 9)	5 (3, 8)	6 (3, 10)
	Anion gap (anion)	Baseline	13 (11, 15)	13 (11, 15)	13 (11, 15)
		Peak	14 (12, 16)	14 (12, 16)	14 (12, 16)
		Number of subjects with ≥1 measurement	12583 (97.2%)	5954 (94.5%)	6629 (99.8%)
		Number of measurements	5 (3, 9)	5 (3, 8)	6 (3, 10)

Summary measures are presented as count (percentage) number of subjects with ≥1 measurement and median (interquartile range) for baseline, peak, and number of measurements.

Supplementary Table 2. Detailed results of simulation studies

A. Model 1 (LME with a linear time trend)

A.1 Using baseline measures

	Scenario 1			Scenario 2			Scenario 3		Scenario 4		Scenario 5	
	**Estimated β**	**TPR**	**FPR**	**Estimated β**	**TPR**	**FPR**	**Estimated β**	**TPR**	**Estimated β**	**FPR**	**Estimated β**	**FPR**
**Group 1**												
Biomarker 1	0.1439 (0.0533)	100.0%	N/A	0.0022 (0.0363)	N/A	10.0%	0.2610 (0.0980)	100.0%	-0.0001 (0.0113)	14.00%	-0.0013 (0.0104)	15.50%
Biomarker 2	0.1451 (0.0601)	100.0%	N/A	0.0011 (0.0295)	N/A	10.0%	0.2632 (0.0977)	100.0%	0.0001 (0.0103)	14.50%	-0.0003 (0.0098)	16.00%
**Group 2**												
Biomarker 3	0.0074 (0.0420)	N/A	6.5%	0.1526 (0.0611)	100.0%	N/A	0.2740 (0.0870)	100.0%	-0.0019 (0.0119)	15.00%	0.0005 (0.0125)	14.50%
Biomarker 4	-0.0044 (0.0392)	N/A	6.5%	0.1517 (0.0601)	100.0%	N/A	0.2715 (0.0831)	100.0%	0.0006 (0.0119)	16.00%	0.0006 (0.0125)	14.50%

A.2 Using peak measures

	Scenario 1			Scenario 2			Scenario 3		Scenario 4		Scenario 5	
	**Estimated β**	**TPR**	**FPR**	**Estimated β**	**TPR**	**FPR**	**Estimated β**	**TPR**	**Estimated β**	**FPR**	**Estimated β**	**FPR**
**Group 1**												
Biomarker 1	0.2012 (0.0853)	100.0%	N/A	-0.0008 (0.0233)	N/A	5.0%	0.1926 (0.0955)	100.0%	-0.0017 (0.0115)	18.00%	-0.0006 (0.0103)	16.00%
Biomarker 2	0.1656 (0.0738)	100.0%	N/A	0.0006 (0.0215)	N/A	5.0%	0.2418 (0.1338)	100.0%	0.0003 (0.0116)	17.50%	-0.0007 (0.0101)	16.50%
**Group 2**												
Biomarker 3	0.0037 (0.0542)	N/A	12.0%	0.1629 (0.0541)	100.0%	N/A	0.2566 (0.0841)	100.0%	0.0001 (0.0124)	17.00%	-0.0012 (0.0112)	13.50%
Biomarker 4	-0.0059 (0.0574)	N/A	12.0%	0.1607 (0.0461)	100.0%	N/A	0.2598 (0.0868)	100.0%	0.0020 (0.0133)	17.00%	0.0006 (0.0114)	13.50%

A.3 Using FPC scores

	Scenario 1			Scenario 2			Scenario 3		Scenario 4		Scenario 5	
	**Estimated β**	**TPR**	**FPR**	**Estimated β**	**TPR**	**FPR**	**Estimated β**	**TPR**	**Estimated β**	**FPR**	**Estimated β**	**FPR**
**Group 1**												
Biomarker 1 FPC1	0.1315 (0.0328)	100.0%	N/A	0.0000 (0.0000)	N/A	0.0%	0.0908 (0.0304)	100.0%	-5e-04 (0.0038)	9.00%	-7e-04 (0.0054)	9.50%
Biomarker 1 FPC2	-0.0125 (0.0428)	94.0%	N/A	0.0000 (0.0000)	N/A	0.0%	-0.0105 (0.0169)	92.0%	0e+00 (0.0051)	9.00%	3e-04 (0.0054)	9.50%
Biomarker 1 FPC3	0.0041 (0.0311)	90.5%	N/A	0.0000 (0.0000)	N/A	0.0%	0.0021 (0.0130)	92.5%	2e-04 (0.0053)	9.00%	-3e-04 (0.0052)	9.50%
Biomarker 2 FPC1	-0.1317 (0.0380)	100.0%	N/A	0.0000 (0.0000)	N/A	0.0%	-0.0934 (0.0539)	100.0%	1e-04 (0.0038)	8.50%	3e-04 (0.0057)	9.50%
Biomarker 2 FPC2	0.0085 (0.0317)	93.5%	N/A	0.0000 (0.0000)	N/A	0.0%	0.0061 (0.0444)	93.5%	8e-04 (0.0052)	9.00%	3e-04 (0.0048)	9.50%
Biomarker 2 FPC3	-0.0047 (0.0291)	90.5%	N/A	0.0000 (0.0000)	N/A	0.0%	-0.0013 (0.0336)	88.0%	0e+00 (0.0035)	9.00%	-3e-04 (0.0038)	9.00%
**Group 2**												
Biomarker 3 FPC1	0.0000 (0.0033)	N/A	3.5%	0.1333 (0.0248)	100.0%	N/A	0.1526 (0.0285)	100.0%	-1e-04 (0.0017)	5.50%	-2e-04 (0.0023)	8.50%
Biomarker 3 FPC2	0.0002 (0.0022)	N/A	3.0%	-0.0157 (0.0232)	92.5%	N/A	-0.0117 (0.0269)	91.5%	-3e-04 (0.0038)	5.50%	0e+00 (0.0031)	8.50%
Biomarker 3 FPC3	-0.0003 (0.0032)	N/A	3.5%	0.0073 (0.0234)	91.0%	N/A	0.0042 (0.0221)	93.0%	0e+00 (0.0026)	5.50%	1e-04 (0.0023)	8.50%
Biomarker 4 FPC1	0.0002 (0.0047)	N/A	3.5%	-0.1343 (0.0234)	100.0%	N/A	-0.1535 (0.0267)	100.0%	3e-04 (0.0028)	5.50%	-2e-04 (0.0044)	8.50%
Biomarker 4 FPC2	-0.0001 (0.0017)	N/A	3.5%	-0.0117 (0.0251)	89.5%	N/A	-0.0127 (0.0257)	93.0%	0e+00 (0.0023)	5.50%	-3e-04 (0.0051)	8.50%
Biomarker 4 FPC3	-0.0002 (0.0023)	N/A	3.5%	0.0069 (0.0216)	91.5%	N/A	0.0047 (0.0247)	87.0%	2e-04 (0.0031)	5.50%	3e-04 (0.0035)	8.50%

B. Model 1 (LME with a quadratic term for time)

B.1 Using baseline measures

	Scenario 1			Scenario 2			Scenario 3		Scenario 4		Scenario 5	
	Estimated β	TPR	FPR	Estimated β	TPR	FPR	Estimated β	TPR	Estimated β	FPR	Estimated β	FPR
**Group 1**												
Biomarker 1	0.1563 (0.0659)	100.0%	N/A	-0.0009 (0.0173)	N/A	21.0%	0.0763 (0.0562)	99.0%	2e-04 (0.0087)	15.50%	6e-04 (0.0092)	17.00%
Biomarker 2	0.1572 (0.0727)	100.0%	N/A	0.0013 (0.0198)	N/A	21.0%	0.0757 (0.0568)	99.0%	0e+00 (0.0083)	15.50%	1e-04 (0.0085)	16.50%
**Group 2**												
Biomarker 3	0.0005 (0.0270)	N/A	18.0%	0.1627 (0.0719)	100.0%	N/A	0.0988 (0.0420)	100.0%	-8e-04 (0.0096)	17.00%	0e+00 (0.0122)	18.00%
Biomarker 4	0.0025 (0.0263)	N/A	18.0%	0.1632 (0.0729)	100.0%	N/A	0.1030 (0.0478)	100.0%	2e-04 (0.0110)	17.50%	-2e-04 (0.0114)	18.00%

B.2 Using peak measures

	Scenario 1			Scenario 2			Scenario 3		Scenario 4		Scenario 5	
	Estimated β	TPR	FPR	Estimated β	TPR	FPR	Estimated β	TPR	Estimated β	FPR	Estimated β	FPR
**Group 1**												
Biomarker 1	0.1535 (0.0642)	100.0%	N/A	-0.0032 (0.0182)	N/A	22.0%	0.0639 (0.0485)	98.0%	7e-04 (0.0085)	14.00%	0.0000 (0.0089)	17.00%
Biomarker 2	0.1539 (0.0658)	100.0%	N/A	-0.0033 (0.0192)	N/A	22.0%	0.0648 (0.0503)	98.0%	8e-04 (0.0088)	13.50%	0.0002 (0.0083)	17.00%
**Group 2**												
Biomarker 3	-0.0020 (0.0263)	N/A	17.0%	0.1626 (0.0723)	100.0%	N/A	0.0902 (0.0387)	100.0%	-8e-04 (0.0104)	17.50%	0.0003 (0.0105)	20.50%
Biomarker 4	0.0008 (0.0255)	N/A	17.0%	0.1638 (0.0747)	100.0%	N/A	0.0945 (0.0433)	100.0%	0e+00 (0.0119)	18.00%	-0.0012 (0.0119)	20.50%

B.3 Using FPC scores

	Scenario 1			Scenario 2			Scenario 3		Scenario 4		Scenario 5	
	Estimated β	TPR	FPR	Estimated β	TPR	FPR	Estimated β	TPR	Estimated β	FPR	Estimated β	FPR
**Group 1**												
Biomarker 1 FPC1	-0.1183 (0.0184)	100.0%	N/A	0.0000 (0.0000)	N/A	0.0%	-0.0357 (0.0180)	98.5%	-1e-04 (0.0041)	12.00%	-1e-04 (0.0021)	4.50%
Biomarker 1 FPC2	0.0107 (0.0300)	98.0%	N/A	0.0000 (0.0000)	N/A	0.0%	-0.0020 (0.0134)	95.0%	0e+00 (0.0027)	12.00%	3e-04 (0.0024)	4.50%
Biomarker 1 FPC3	-0.0011 (0.0140)	90.0%	N/A	0.0000 (0.0000)	N/A	0.0%	-0.0007 (0.0073)	87.5%	3e-04 (0.0039)	11.50%	1e-04 (0.0017)	4.50%
Biomarker 2 FPC1	-0.1186 (0.0215)	100.0%	N/A	0.0000 (0.0000)	N/A	0.0%	-0.0374 (0.0313)	98.5%	-4e-04 (0.0032)	12.00%	-1e-04 (0.0007)	4.00%
Biomarker 2 FPC2	0.0015 (0.0317)	96.0%	N/A	0.0000 (0.0000)	N/A	0.0%	-0.0012 (0.0351)	89.5%	4e-04 (0.0057)	12.00%	-1e-04 (0.0020)	4.50%
Biomarker 2 FPC3	-0.0003 (0.0140)	87.5%	N/A	0.0000 (0.0000)	N/A	0.0%	-0.0007 (0.0073)	87.0%	3e-04 (0.0036)	11.50%	-1e-04 (0.0024)	4.50%
**Group 2**												
Biomarker 3 FPC1	-0.0003 (0.0039)	N/A	0.5%	-0.1148 (0.0128)	100.0%	N/A	-0.0662 (0.0128)	100.0%	-1e-04 (0.0036)	10.50%	1e-04 (0.0024)	5.00%
Biomarker 3 FPC2	-0.0004 (0.0057)	N/A	0.5%	0.0208 (0.0297)	97.0%	N/A	0.0011 (0.0213)	95.0%	-4e-04 (0.0044)	10.50%	-4e-04 (0.0022)	5.00%
Biomarker 3 FPC3	-0.0001 (0.0011)	N/A	0.5%	0.0039 (0.0136)	90.0%	N/A	-0.0001 (0.0132)	90.0%	4e-04 (0.0047)	10.50%	-1e-04 (0.0028)	5.00%
Biomarker 4 FPC1	0.0004 (0.0063)	N/A	0.5%	-0.1147 (0.0126)	100.0%	N/A	-0.0684 (0.0181)	100.0%	-2e-04 (0.0037)	10.50%	3e-04 (0.0019)	5.00%
Biomarker 4 FPC2	0.0002 (0.0033)	N/A	0.5%	0.0192 (0.0295)	97.0%	N/A	0.0019 (0.0204)	90.5%	3e-04 (0.0037)	10.00%	0e+00 (0.0023)	5.00%
Biomarker 4 FPC3	-0.0002 (0.0021)	N/A	0.5%	0.0053 (0.0142)	89.0%	N/A	-0.0001 (0.0104)	92.5%	2e-04 (0.0035)	10.50%	1e-04 (0.0030)	5.00%

C. Model 1 (LME with a 3-knot spline function for time)

C.1 Using baseline measures

	Scenario 1			Scenario 2			Scenario 3		Scenario 4		Scenario 5	
	Estimated β	TPR	FPR	Estimated β	TPR	FPR	Estimated β	TPR	Estimated β	FPR	Estimated β	FPR
**Group 1**												
Biomarker 1	0.1327 (0.0479)	100.0%	N/A	-0.0003 (0.0224)	N/A	5.5%	0.2131 (0.1193)	98.5%	-8e-04 (0.0095)	16.50%	-0.0003 (0.0096)	20.00%
Biomarker 2	0.1383 (0.0433)	100.0%	N/A	-0.0013 (0.0265)	N/A	5.5%	0.2203 (0.1239)	98.5%	9e-04 (0.0110)	17.50%	0.0011 (0.0106)	19.50%
**Group 2**												
Biomarker 3	-0.0007 (0.0190)	N/A	5.5%	0.1429 (0.0516)	100.0%	N/A	0.2459 (0.0992)	100.0%	-7e-04 (0.0130)	16.00%	-0.0002 (0.0147)	16.00%
Biomarker 4	0.0017 (0.0206)	N/A	5.5%	0.1413 (0.0468)	100.0%	N/A	0.2440 (0.0952)	100.0%	2e-04 (0.0135)	16.00%	-0.0005 (0.0146)	16.00%

C.2 Using peak measures

	Scenario 1			Scenario 2			Scenario 3		Scenario 4		Scenario 5	
	Estimated β	TPR	FPR	Estimated β	TPR	FPR	Estimated β	TPR	Estimated β	FPR	Estimated β	FPR
**Group 1**												
Biomarker 1	0.1970 (0.0856)	100.0%	N/A	0.0044 (0.0273)	N/A	5.0%	0.2350 (0.1384)	99.5%	0.0150 (0.0230)	41.50%	0.0013 (0.0115)	19.50%
Biomarker 2	0.1584 (0.0658)	100.0%	N/A	-0.0047 (0.0282)	N/A	5.0%	0.1880 (0.1106)	99.5%	-0.0020 (0.0183)	40.50%	0.0005 (0.0119)	20.00%
**Group 2**												
Biomarker 3	0.0207 (0.0702)	N/A	10.0%	0.1883 (0.0699)	100.0%	N/A	0.3113 (0.1489)	100.0%	0.0148 (0.0280)	40.50%	-0.0005 (0.0148)	16.50%
Biomarker 4	-0.0202 (0.0719)	N/A	10.0%	0.1415 (0.0339)	100.0%	N/A	0.1781 (0.0755)	100.0%	-0.0157 (0.0295)	40.50%	-0.0008 (0.0135)	16.50%

C.3 Using FPC scores

	Scenario 1			Scenario 2			Scenario 3		Scenario 4		Scenario 5	
	Estimated β	TPR	FPR	Estimated β	TPR	FPR	Estimated β	TPR	Estimated β	FPR	Estimated β	FPR
**Group 1**												
Biomarker 1 FPC1	0.1215 (0.0335)	100.0%	N/A	0.0000 (0.0000)	N/A	0.0%	0.0901 (0.0638)	99.0%	2e-04 (0.0037)	9.00%	-1e-04 (0.0028)	6.50%
Biomarker 1 FPC2	-0.0025 (0.0337)	95.0%	N/A	0.0000 (0.0000)	N/A	0.0%	0.0025 (0.0502)	89.5%	1e-04 (0.0041)	8.50%	-1e-04 (0.0028)	6.50%
Biomarker 1 FPC3	0.0086 (0.0294)	91.0%	N/A	0.0000 (0.0000)	N/A	0.0%	0.0025 (0.0269)	89.0%	2e-04 (0.0037)	8.50%	0e+00 (0.0040)	6.50%
Biomarker 2 FPC1	-0.1251 (0.0336)	100.0%	N/A	0.0000 (0.0000)	N/A	0.0%	-0.0936 (0.0777)	99.0%	-3e-04 (0.0033)	8.50%	-2e-04 (0.0036)	5.50%
Biomarker 2 FPC2	-0.0130 (0.0306)	94.0%	N/A	0.0000 (0.0000)	N/A	0.0%	-0.0182 (0.0723)	93.5%	-3e-04 (0.0027)	8.50%	1e-04 (0.0041)	6.00%
Biomarker 2 FPC3	-0.0010 (0.0299)	95.5%	N/A	0.0000 (0.0000)	N/A	0.0%	-0.0071 (0.0605)	90.5%	-1e-04 (0.0035)	8.50%	2e-04 (0.0038)	6.50%
**Group 2**												
Biomarker 3 FPC1	0.0000 (0.0028)	N/A	3.5%	0.1337 (0.0297)	100.0%	N/A	0.1552 (0.0464)	100.0%	-1e-04 (0.0035)	8.00%	2e-04 (0.0030)	5.50%
Biomarker 3 FPC2	0.0000 (0.0049)	N/A	4.0%	-0.0061 (0.0279)	94.5%	N/A	-0.0031 (0.0291)	93.5%	4e-04 (0.0040)	8.00%	3e-04 (0.0033)	5.00%
Biomarker 3 FPC3	-0.0001 (0.0042)	N/A	4.0%	0.0077 (0.0229)	88.5%	N/A	0.0102 (0.0247)	88.5%	3e-04 (0.0033)	8.00%	4e-04 (0.0036)	5.50%
Biomarker 4 FPC1	0.0002 (0.0029)	N/A	4.0%	-0.1289 (0.0263)	100.0%	N/A	-0.1562 (0.0624)	100.0%	0e+00 (0.0029)	8.00%	0e+00 (0.0028)	5.50%
Biomarker 4 FPC2	-0.0002 (0.0035)	N/A	4.0%	-0.0112 (0.0266)	96.5%	N/A	-0.0190 (0.0663)	92.5%	-1e-04 (0.0026)	7.50%	2e-04 (0.0032)	5.00%
Biomarker 4 FPC3	-0.0002 (0.0021)	N/A	4.0%	-0.0005 (0.0227)	88.0%	N/A	0.0008 (0.0296)	88.5%	-1e-04 (0.0024)	7.50%	0e+00 (0.0030)	5.50%

Mean and standard deviation of estimated β across 200 repetitions, true positive rate (TPR) calculated as the proportion of simulations where truly non-zero coefficients were selected, or false positive rate (FPR) calculated as the proportion of simulations where truly zero coefficients were selected
