# Supplementary material for: Functional principal component analysis and sparse-group LASSO to identify associations between biomarker trajectories and mortality among hospitalized SARS-CoV-2 infected individuals
Source: BMC Med Res Methodol. 2023 Oct 28;23:254. doi: 10.1186/s12874-023-02076-3 (PMC10613396; doi:10.1186/s12874-023-02076-3)
Supplement: Supplementary file 2 — Additional file 2. Supplementary Methods. [file 12874_2023_2076_MOESM2_ESM.pdf]

This Supplementary Methods section provides a detailed description on how the simulation studies were set up.

We first simulated the true trajectories for four biomarkers under three models:

Model 1. A linear mixed-effects model (LME) with a linear time trend:

$$Z_{ki}(t) = \beta_{k0} + \beta_{k1} \times t + \gamma_{ki0} + \gamma_{ki1} \times t$$

where the fixed effect coefficients were

k	1	2	3	4
Intercepts β_{k0}	0.5	-0.8	0.2	-0.5
Slopes β_{k1}	3	-1	1	1

and the random effect coefficients followed distributions:

$$\begin{pmatrix} \gamma_{1i0} \\ \gamma_{2i0} \\ \gamma_{1i1} \\ \gamma_{2i1} \end{pmatrix} \overset{\text{iid}}{\sim} \text{MVN}_4 \left(\mathbf{0}, \mathbf{\Sigma}_{\text{low}} = 0.3^2 \begin{pmatrix} 1 & 0.2 & 0.1 & 0.1 \\ 0.2 & 1 & 0.1 & 0.1 \\ 0.1 & 0.1 & 1 & 0.2 \\ 0.1 & 0.1 & 0.2 & 1 \end{pmatrix} \right)$$

$$\begin{pmatrix} \gamma_{3i0} \\ \gamma_{4i0} \\ \gamma_{3i1} \\ \gamma_{4i1} \end{pmatrix} \overset{\text{iid}}{\sim} \text{MVN}_4 \left(\mathbf{0}, \mathbf{\Sigma}_{\text{high}} = 0.3^2 \begin{pmatrix} 1 & 0.8 & 0.1 & 0.1 \\ 0.8 & 1 & 0.1 & 0.1 \\ 0.1 & 0.1 & 1 & 0.8 \\ 0.1 & 0.1 & 0.8 & 1 \end{pmatrix} \right)$$

for $i = 1, 2, \cdots, n$

Model 2. An LME with a quadratic term for time:

$$Z_{ki}(t) = \beta_{k0} \times (t - \beta_{k1})^2 + \gamma_{ki0} + \gamma_{ki1} \times t$$

where the fixed effect coefficients were

k	1	2	3	4
β_{k0}	-0.1	-0.4	-0.2	-0.2
β_{k1}	0.1	0.12	0.08	0.07

and the random effect coefficients followed the same distributions as in Model 1.

Model 3. An LME with a 3-knot spline function for time:

$$Z_{ki}(t) = \beta_{k0} + \beta_{k1} \times t + \sum_{q=1}^{3} b_{kq}(t - \tau_{kq})_+ + \gamma_{ki0} + \gamma_{ki1} \times t$$

where the fixed effect coefficients were

k	1	2	3	4
Intercepts β_{k0}	0.5	-0.8	0.2	-0.5
Slopes β_{k1}	3	-1	1	1
b_{k1}	-2	1.5	0.5	-1.5
b_{k2}	-2	0.5	-1	1.5
b_{k3}	3	-1	-1	1.5

and the random effect coefficients followed the same distributions as in Model 1.

Next, we simulated death times based on the true biomarker trajectories using inverse transform sampling on the survival function derived from the following hazard function:

$$h_i(t) = h_0(t)\text{exp}\left[\alpha_1 \times Z_{1i}(t) + \alpha_2 \times Z_{2i}(t) + \alpha_3 \times Z_{3i}(t) + \alpha_4 \times Z_{4i}(t)\right]$$

where the baseline Weibull hazard function was defined as $h_0(t) = 2te^{-2}$ for biomarker trajectories simulated under Model 1 and Model 3, and as $h_0(t) = 2te^{2}$ for biomarker trajectories simulated under Model 2. A censoring time t_{cen} was picked to ensure that at least 200 (10%) deaths were observed. Regarding the values of the association parameters $\alpha_1, \alpha_2, \alpha_3, \alpha_4$, we constructed 4 scenarios for each of the 3 models: in Scenario 1, only the biomarker group with low correlation was associated with mortality rates, thus $\alpha_1 = \alpha_2 = 1, \alpha_3 = \alpha_4 = 0$; in Scenario 2, only the biomarker group with high correlation was associated with mortality rates, thus $\alpha_1 = \alpha_2 = 0, \alpha_3 = \alpha_4 = 1$; in Scenario 3, both 2 biomarker groups were associated with mortality rates, thus $\alpha_1 = \alpha_2 = \alpha_3 = \alpha_4 = 1$ for Model 1 and Model 3 and $\alpha_1 = \alpha_2 = \alpha_3 = \alpha_4 = 0.5$ for Model 2; in Scenario 4 which was the null case, neither of the 2 biomarker groups was associated with mortality rates, thus $\alpha_1 = \alpha_2 = \alpha_3 = \alpha_4 = 0$.

Lastly, we simulated observed trajectories for the four biomarkers. Each patient had a baseline measurement at $t = 0$ and 9 other measurements at time simulated independently from a truncated exponential distribution with rate parameter $3/t_{\text{cen}}$ and maximum at t_{cen}. Measurement times were rounded to one decimal place and then duplicated time points for each individual were removed such that we acquired a set of sparse and irregular longitudinal measurements. Then we simulated noise as measurement errors to add to the true trajectories:

$$Z'_{ki}(t) = Z_{ki}(t) + \epsilon_{kit},\ \epsilon_{kit} \overset{\text{iid}}{\sim} \text{N}(0, 0.1^2) \text{ for } k = 1,2,3,4,\ i = 1,2,\cdots,n,\ ,t = 1,2,\cdots,10$$

We also simulated patients' discharge time independently from a Gamma distribution with shape parameter 2 and scale parameter $\frac{3}{8}t_{\text{cen}}$. Then we censored observed biomarker trajectories at each patient's death time or discharge time.
